# Reactivity to Interpersonal Conflict: A Correlate of Non‐Remission in Childhood Depression—Evidence Across Modalities and Time Scales

**DOI:** 10.1155/da/5803578

**Published:** 2026-07-28

**Authors:** Deanna M. Barch, Laura Hennefield, Renee J. Thompson, Caroline P. Hoyniak, Rebecca Tillman, Katherine Luking, Margaret M. Redic, Kirsten Gilbert, Meghan Rose Donohue, Diana Whalen, Joan L. Luby

**Affiliations:** ^1^ Department of Psychiatry, Washington University School of Medicine, St. Louis, Missouri, USA, wustl.edu; ^2^ Department of Psychological and Brain Sciences, Washington University, St. Louis, Missouri, USA, wustl.edu; ^3^ The Program in Neuroscience, Washington University, St. Louis, Missouri, USA, wustl.edu; ^4^ Department of Radiology, Washington University School of Medicine, St. Louis, Missouri, USA, wustl.edu; ^5^ Department of Psychology, Saint Louis University, St. Louis, Missouri, USA, slu.edu

**Keywords:** depression, family conflict, interpersonal, peer conflict, rejection, relapse

## Abstract

Depression is often a relapsing disorder that can start as early as preschool. In adults and adolescents, family and peer criticism/conflict and rejection sensitivity are associated with recurrence. However, it is not yet clear whether similar factors are associated with relapse among early adolescent youth who experience depression starting in preschool. The current study used data from a unique longitudinal study, which began at preschool age and included 161 youth. It oversampled for those with preschool‐onset major depressive disorder (PO‐MDD) and included community controls (CCs). When the youth were 7–12 years old, we administered diagnostic interviews and weekly surveys over 1 year. These measures were repeated at ages 8–15, along with ecological momentary assessment (EMA) three to four times a day for 8 days, and an event‐related potential (ERP) task assessing neural reactivity to social feedback. We assessed whether youth with a history of PO‐MDD who were experiencing depression in adolescence (i.e., non‐remitted youth [NRY]) versus those who were not (i.e., remitted youth [RY]) and/or CCs experienced greater family/peer criticism/conflict and sensitivity to interpersonal stress. Across multiple time scales (weekly and daily), NRY experienced greater parent and peer criticism/conflict and were more reactive to such criticism/conflict than RY and CCs. Further, in weekly surveys, NRY displayed a stronger association between experiencing interpersonal criticism/conflict and depression compared to CCs, with a similar trend compared to RY. In momentary assessments, NRY showed a longer‐lasting association between family conflict and both negative affect (NA) and connectedness than RY. In addition, neural measures indicated a stronger response to negative social feedback among NRY. Taken together, these data identify potential interpersonal treatment targets for youth with early‐onset depression who continue to experience depression into adolescence.

**Trial Registration:** ClinicalTrials.gov identifier: NCT02076425

## 1. Introduction

Depression is a chronic and relapsing disorder that can start as early as preschool (i.e., preschool‐onset major depressive disorder [PO‐MDD]). Predictors of remission versus relapse into adolescence are critical to treatment planning for PO‐MDD. In research examining depression that onsets in older adolescence and adulthood, several key factors have been associated with remission versus relapse, including interpersonal factors such as family or peer criticism/conflict [1–3] and rejection sensitivity [4]. The finding that PO‐MDD predicts early adolescent MDD has been established [5], and longitudinal studies of PO‐MDD show both homotypic and heterotypic continuity [6–8]. However, these studies did not examine the interpersonal functioning in youth with a history of PO‐MDD, and whether these factors operate similarly in this population remains unknown. This is particularly important as these interpersonal patterns are more malleable earlier in development and may therefore be a missed treatment target. Thus, the current study used data from a unique longitudinal study, which began at the preschool age and oversampled youth who had PO‐MDD.

We had several aims. First, we examined whether youth with PO‐MDD who experienced depression again in adolescence, versus those who did not or community controls (CCs), had greater family/peer criticism/conflict, self‐reported rejection sensitivity, depression, suicidal ideation (SI), and negative affect (NA), as well as lower positive affect (PA). Second, we examined whether youth with PO‐MDD who experienced depression again in adolescence showed stronger associations of family/peer criticism/conflict with depression symptoms, NA, SI, and PA versus youth with PO‐MDD who did not or controls. Third, we examined the direction of the associations between family and peer criticism/conflict and depression and affective disturbances to determine whether family/peer criticism/conflict contributed to affective disturbances or whether youth experiencing affective disturbances might elicit more interpersonal criticism/conflict. We examined these aims using intensive longitudinal methods assessing constructs of interest at multiple time scales, including weekly and momentary, which have yet to be done in PO‐MDD. Fourth, to further investigate the role of reactivity to interpersonal negativity, we also examined neural responses to social feedback using event‐related potentials (ERPs). Understanding the role of these factors is critical for identifying and informing novel treatment targets for those who may have more chronic, unremitting courses of depression and better identifying those who may benefit from booster sessions after early intervention.

Parent‐child criticism/conflict is a key risk factor in MDD during adolescence [1]. Mothers of youth with MDD have higher expressed criticism toward their children than mothers of nondepressed youth [9]. Further, high levels of maternal expressed criticism predicted risk for depression in youth over a 14‐month follow‐up [10]. There is also evidence that greater perceived maternal hostility and lower perceived maternal warmth are associated with higher rates of depression in offspring, particularly for mothers also experiencing MDD [11]. Such perceptions of maternal hostility may either reflect actual greater hostility or a greater sensitivity to hostility among youth at risk for depression. In addition, measures of parental expressed emotion, which include criticism and emotional overinvolvement, are higher in families of depressed children versus those with schizophrenia spectrum disorders [12], predict greater persistence of depression in children [13], and predict increases in depression over 1 year [14]. Furthermore, parent‐child criticism/conflict in adolescents with depression is a key risk factor for adult depression [15]. There is evidence of bidirectional effects, with child psychopathology also predicting maternal criticism over time [16, 17]. However, it is unclear whether youth with a history of PO‐MDD who have depression in adolescence experience higher rates of parental criticism/conflict or criticism relative to youth with PO‐MDD who did not have depression in adolescence. If so, it will also be important to investigate whether this is driven by (a) greater reactivity to interpersonal negativity (e.g., they experience greater and more enduring NA or depressive symptoms in response to parental criticism or conflict) and/or (b) eliciting more parental criticism/conflict due to ongoing reactivity to interpersonal negativity.

Conflict with peers is another interpersonal factor that may be salient in understanding ongoing experiences of depression in adolescents with a history of PO‐MDD. Among adolescents, peer support has been identified as a key protective factor for depression [18], whereas peer victimization (e.g., social exclusion and target of physical aggression) is a risk factor for depression [19–21]. Other work has highlighted conflicts with friends or classmates as a risk factor for depression in adolescents [2, 3], and network analysis of risk pathways for adolescent depression has demonstrated that peer conflict is distinct from family conflict [22]. However, it is unclear whether peer criticism/conflict is associated with the recurrence of depression in youth with a history of PO‐MDD.

Crucially, how sensitive or reactive youth are to interpersonal criticism/conflict, such as parent and peer criticism/conflict, may be an important risk factor for non‐remission or relapse, above the frequency and severity of such criticism/conflict. Rejection sensitivity, frequently defined as the tendency to anxiously expect, readily perceive, and intensely react to social rejection [4, 23, 24], has been identified as a significant risk factor for relapse in MDD during adolescence and early adulthood [4, 23, 24]. This heightened sensitivity can exacerbate emotional responses to real or perceived social slights, contributing to the recurrence of depressive episodes. This type of rejection sensitivity could be a trait‐like characteristic that renders some individuals more vulnerable to depression or a mediating mechanism by which chronic conflict with family or peers contributes to or exacerbates depression. For example, Downey and Feldman [58] found that individuals with high rejection sensitivity were more likely to perceive and overreact to social rejection, which could heighten depressive symptoms and create a higher risk for relapse. Further, increased engagement and attention to negative social feedback have been associated with depression [25]. Relatedly, rejection sensitivity in late adolescence predicted increases in depressive symptoms and anxiety over 3 years [4], and other work has highlighted an association between sensitivity to peer rejection and depression in adolescents [26]. Thus, it is possible that youth with a history of PO‐MDD who have depression in adolescence might be more sensitive to interpersonal rejection and criticism/conflict, leading even relatively normative experiences of interpersonal criticism/conflict to have an outsized effect on NA and depressive symptoms.

There is also neural evidence that individuals with or at risk for depression show increased sensitivity to social feedback. For example, female adolescents at risk for depression by virtue of having a mother with MDD show (a) greater increases in self‐reported depression and social disconnection [27], (b) greater amygdala activation following social evaluation [27], and (c) greater anterior insula activation to social feedback [28]. In addition, adults with MDD exhibit greater amygdala, insula, and ventrolateral prefrontal cortex activation in response to social rejection [29], as well as increased insula responses to social feedback compared to healthy controls [30]. Further, adolescents with depression versus their non‐depressed peers show increased amygdala, subgenual ACC and anterior insula, and accumbens activation to social rejection [31].

Neural sensitivity to social feedback has also been examined using ERPs, focusing on a component referred to as the reward positivity (RewP) [32]. The RewP reflects the difference in responses to acceptance versus rejection feedback, with a more positive‐going response to social acceptance and a more negative‐going response to social rejection. There is some evidence that adolescents with depression, particularly those with heightened rejection sensitivity [33], show a blunted response to social acceptance feedback [32, 34, 35]. However, it is less clear that greater responsivity to social rejection feedback assessed using ERPs might also be related to depression. Thus, research is needed to determine whether youth with PO‐MDD who experience depression again in adolescence might show stronger responses to social rejection feedback and/or blunted responses to social acceptance.

To address the questions outlined above, we used data from a longitudinal study of children who underwent treatment for PO‐MDD with parent–child interaction therapy–emotion development (PCIT‐ED), as well as CCs, who were followed up approximately 4 years later. Importantly, within the study, all youth with PO‐MDD received the active treatment either immediately or after a waiting period. These youth participated in multiple intensive longitudinal assessment waves of data collection, assessing constructs of interest at weekly/monthly and momentary timeframes. First, for one to 2 years, they completed weekly or monthly assessments of parent and peer criticism/conflict, the need to belong, perceived burdensomeness, depression, affect, and SI during the pre‐ and early‐adolescent period. Second, they completed ecological momentary assessment (EMA) surveys of similar constructs at the momentary level for eight consecutive days. They also participated in an ERP session to assess neural responses to social feedback prior to the start of the EMA. Thus, we assessed our constructs of interest with different approaches (i.e., intensive longitudinal and ERP) and time scales (i.e., weekly and momentary), providing converging evidence.

Our first goal was to test the hypothesis that youth with PO‐MDD who experienced depression in adolescence (i.e., non‐remitted youth [NRY]) would report higher rates of parent and peer criticism/conflict, greater feelings of rejection sensitivity (burdensomeness and thwarted belonging), depression, SI, and NA, and lower PA compared to remitted youth (RY) or CCs. Our second goal was to test the hypothesis that NRY would show a stronger association of parent/peer criticism/conflict and rejection sensitivity to depression, affect, and SI compared to RY and CCs. Our third goal was to examine the direction of the associations between family/peer criticism/conflict and depression and affective disturbances to help determine if NRY are more reactive to family/peer criticism/conflict or more likely to elicit interpersonal criticism/conflict. To do so, we examined temporally lagged analyses to determine whether family or peer criticism/conflict predicted later affect disturbances and/or depressive symptoms or whether depression/affective disturbances predicted later family or peer criticism/conflict. We also examined whether either of these directions of association was stronger among NRY compared to RY. Our fourth goal was to test the hypothesis that NRY would show blunted neural responses to positive social feedback (i.e., acceptance) and stronger neural responses to negative peer feedback (i.e., rejection) compared to RY and CCs. If hypotheses are supported, it would point to key interpersonal areas of focus for booster treatments for youth with PO‐MDD who experience depression again in adolescence.

## 2. Materials and Methods

### 2.1. Participants

This study is a multi‐visit, longitudinal follow‐up to a randomized controlled trial (RCT) for PO‐MDD, titled PCIT‐ED[36, 37]. To be eligible for the current study, youth needed to complete the baseline assessment of PCIT‐ED or be a CC from the PCIT‐ED study (*N* = 368). These youth were asked to complete a follow‐up assessment between the ages of 7–12 years (Wave 1) and a second follow‐up between the ages of 8–15 years (Wave 2).

For the purposes of examining correlates of remission status, we created three groups of participants: (a) CCs who had no lifetime history of suicidal thoughts or behaviors through their final wave of data collection (CC group; *n* = 40, 8 with Wave 1 as final); (b) youth with PO‐MDD who were not experiencing MDD or MDD‐not otherwise specified (MDD‐NOS) at the time of their final follow‐up assessment and had at least a 50% reduction in MDD severity from the pre‐therapy assessment to their final follow‐up assessment (RY; *n* = 77, 21 with Wave 1 as final); and (c) youth with PO‐MDD who were experiencing MDD or MDD‐NOS at the time of their final follow‐up or did not have at least a 50% reduction in MDD severity from pretherapy to their final follow‐up (NRY; *n* = 44, 11 with Wave 1 as final). Examples of youth excluded from these analyses include CCs who developed STBs, youth at baseline with subclinical symptoms or who were not randomized into the trial due to suspected ASD or developmental delays, or youth who were not retained. Demographic and clinical characteristics of the 161 participants who completed either follow‐up wave and were included in the current analyses are presented in Table 1(Table S1 presents demographics of included versus not included participants; Table S2 shows Waves 1 and 2 separately). Child assent and caregiver written consent were obtained, and the Institutional Review Board preapproved all procedures prior to data collection (#201306070).

**Table 1:** Demographic and clinical characteristics (*N* = 161).

Subject characteristics	Community control (*N* = 40)		Remitted (*N* = 77)		Non‐ remitted (*N* = 44)		Omnibus test	Remitted vs. community control	Non‐ remitted vs. community control	Remitted vs. non‐ remitted
	Mean	*SD*	Mean	SD	Mean	SD	*p*	*p*	*p*	*p*
Age at final wave	11.74	1.32	11.91	1.41	12.44	1.36	0.0489	0.5200	0.0218	0.0457
Final wave INR	2.78	0.87	3.06	0.81	2.82	1.00	0.1722	—	—	—
N therapy sessions^a^	–	–	17.42	6.05	17.61	5.76	0.8605	—	—	—
Final wave MDD core score	0.48	0.88	0.61	0.71	3.50	1.91	<0.0001	0.5616	<0.0001	<0.0001

	**%**	***n***	**%**	***n***	**%**	***n***	***p***	***p***	***p***	***p***

Female sex	52.5	21	22.1	17	43.2	19	0.0028	0.0008	0.4075	0.0214
Hispanic ethnicity	7.5	3	5.2	4	4.6	2	0.8251	—	—	—
Race^b^	—	—	—	—	—	—	0.0924	—	—	—
White	80.0	32	87.0	67	70.5	31	—	—	—	—
Black	10.0	4	3.9	3	15.9	7	—	—	—	—
Asian	5.0	2	1.3	1	2.3	1	—	—	—	—
Multiracial	5.0	2	7.8	6	11.4	5	—	—	—	—

^a^Community control participants not included.

^b^Race modeled as white versus non‐white.

Abbreviation: INR, income‐to‐needs ratio.

### 2.2. Supporting Materials

The Supporting Information include a figure illustrating the study design, a figure presenting additional results, and additional tables providing further statistical details of the analyses. These tables and figures are referenced in their respective sections below.

### 2.3. Overview of Study Design

A graphic overview of the study design with sample sizes is provided in Figure S1. At each wave, youth were invited to the lab to complete diagnostic interviews, parent and self‐report measures, and an ERP task. Youth also completed a tutorial on how to respond to an “e‐survey” that they were asked to complete either weekly or monthly, depending on their history of SI/behaviors or non‐suicidal self‐injurious behavior (details are given below) for 12 months. At follow‐up Wave 2, youth with a cellphone compatible with the EMA software (with caregiver permission) were asked to participate in a 38‐day EMA and passive sensing component of the study using the Effortless Assessment of Risk States (EARS) [38, 39] mobile app.

### 2.4. Measures

#### 2.4.1. Psychiatric Diagnoses

Psychiatric diagnoses were assessed using the Kiddie Schedule for Affective Disorders and Schizophrenia–Present and Lifetime (K‐SADS‐PL) [40], a semi‐structured diagnostic clinical interview for DSM‐5 disorders. Trained researchers administered the diagnostic interview separately to the caregiver and youth at each follow‐up wave, blind to the remission status. Caregivers reported on their child’s lifetime and current (over the prior month) symptoms. Children reported their current symptoms. Children were considered to meet the criteria for a given symptom if endorsed by either the caregiver or the child. Licensed clinicians served as the master raters and held weekly case conferences. Kappas for MDD/MDD‐NOS across waves were 0.78 and 0.77 for caregiver and child assessments, respectively.

#### 2.4.2. Depressive Symptoms

Depressive symptoms were assessed at the lab session using the K‐SADS‐PL, with MDD core severity score calculated as the number of nine core MDD symptoms endorsed by the caregiver and/or child.

### 2.5. Intensive Longitudinal Methods

#### 2.5.1. Weekly/Monthly At‐Home Assessments

The goal of the e‐surveys was to assess weekly variation in depression symptoms, affect, and suicidal thoughts, as well as to understand potential correlates or precipitants of this variation, including criticism/conflict with family or peers and rejection sensitivity. For the e‐surveys, researchers first guided the youth through an interactive tutorial. Caregivers and youth were informed at multiple time points (e.g., during consent/assent and during the tutorial) that no team member would monitor the responses to the e‐surveys. Researchers encouraged youth to tell a trusted adult immediately if they experienced STBs. A message at the end of each e‐survey reiterated this and provided mental health resources.

Youth completed e‐surveys at a weekly frequency if their caregiver reported STBs or NSSI during the PCIT‐ED study or if they or their caregiver reported lifetime STBs or NSSI at either the Wave 1 or 2 assessment (*n* = 91 of 150 youth at Wave 1 and 81 of 118 youth at Wave 2) or at a monthly frequency if their caregiver did not report STBs or NSSI during PCIT‐ED and if they and their caregiver did not report ever experiencing STBs or NSSI at the current or previous follow‐up wave (*n* = 59 of 150 youth at Wave 1 and 37 of 118 youth at Wave 2). Hyperlinks to e‐surveys were sent to a caregiver’s email or cellphone or the child’s email or cellphone. Youth were compensated $10 for each e‐survey. E‐surveys were administered through the HIPAA‐compliant Research Electronic Data Capture (REDCap) platform [41]. Participants had 2 days (for weekly e‐surveys) or 6 days (for monthly e‐surveys) to complete each e‐survey. Supplemental Methods provides completion rates.

The following constructs were assessed at each e‐survey, with all but one item (“thoughts of killing yourself right now”) instructing participants to consider the timeframe of “the past week.” Participants rated all items except the depressive symptoms and SI items using a visual analog scale from never (0) to all the time (100). Depressive symptom responses used a radio button with three options for each item, and SI responses used a checkbox format. Information on the strong construct validity of these assessments is provided in a previous report assessing Wave 1 e‐survey risk factors and STB reports [42].

##### 2.5.1.1. Depressive Symptoms

Depressive symptoms were assessed through the CDI‐2 [43], omitting two items to reduce redundancy: thinking that bad things happen to me and thinking about killing myself. The three options for each item were the standard CDI‐2 response options. A total score was created by averaging responses at the e‐survey level.

##### 2.5.1.2. Affect

Affect was assessed using a subset of items from the positive affect and NA schedule–child [44]. Mean levels of NA (sad, lonely, bored, frustrated, scared, and nervous) and PA (calm, interested, cheerful, happy, excited, and proud) were computed at the e‐survey level.

##### 2.5.1.3. SI

SI was assessed using the 4‐item ask suicide‐screening questions [45]. Participants were asked to answer yes or no to the original first three items regarding the past week: (a) “Have you wished you were dead?” (b) “Have you felt that you or your family would be better off if you were dead?” and (c) “Have you been having thoughts about killing yourself?” The fourth item was modified from, “Have you ever tried to kill yourself?” to “Are you having thoughts of killing yourself right now?” A binary score (0 = all “no” responses and 1 = one or more “yes” responses) was computed at the e‐survey level.

##### 2.5.1.4. Rejection Sensitivity: Thwarted Belongingness

Thwarted belongingness was assessed using two items from the Interpersonal Needs Questionnaire (INQ‐10) [46]: “I felt disconnected or like I was an outsider” and “I have felt that I had many caring and supportive friends or family members” (reverse scored). Responses were averaged at the e‐survey level.

##### 2.5.1.5. Rejection Sensitivity: Perceived Burdensomeness

Perceived burdensomeness was assessed using one item from the INQ‐10 [46]: “I felt that the people in my life would be better off if I were gone.”

##### 2.5.1.6. Criticism and Conflict

Caregiver‐child and peer criticism and conflict were each assessed with two items from the Perceived Criticism Scale [47]: “How often has/have your (relation) criticized you?” and “How often have you argued with your (relation)?” For caregiver items, the relation was “parent/caregiver(s)” and the peer items were populated with “friends or classmates.” The two caregiver items and the two peer items were each averaged separately at the e‐survey level.

#### 2.5.2. EMA

The goal of the EMA surveys was to assess variation in momentary affect and SI, as well as to understand potential correlates or precipitants of this variation, including conflict with family or peers. Adolescents who agreed to participate in the EMA component of the study (*n* = 53) were taught how to install the EARS mobile app [38, 48] on their smartphones. The app collected EMA data and mobile sensor data (not examined in the current study) for 38 days. The current study used EMA from the first 8 days, during which youth completed surveys to assess their affect, social interactions, and feelings of connectedness multiple times per day. All item responses used a slider going from not at all (0) to extremely (100) (exceptions noted below). Supporting Information provides timing of EMA prompts, training, and payment.

##### 2.5.2.1. Momentary Affect and Connectedness

Youth were asked to rate their momentary affect with 12 questions assessing PA (confident, happy, calm, excited), NA (bored, stressed, sad, and angry), and connectedness (supported, included, rejected [reverse scored], and lonely [reverse scored]). Each survey asked, “How X do you feel right now?” where X was replaced with the specific emotion word in a randomized order. Each item was rated on a scale of 0–100, anchored at not at all to extremely. Items for each subscale were averaged after reverse scoring.

##### 2.5.2.2. Momentary Criticism and Conflict

To assess momentary caregiver‐child and peer criticism and conflict, youth were asked the following questions in a non‐randomized order: (a) “In the past hour, who have you interacted the most with?” (alone, friends, and family); (b) “In the past hour, how much have you had fights or disagreements with your friends?” (rated on a slider of 0–100, anchored at not at all to extremely); and (c) “In the past hour, how much have you had fights or disagreements with your family?” (rated on a slider of 0–100, anchored at not at all to extremely).

To assess SI, participants were asked three questions: (a) “Since the last prompt, how often have you felt that you or your family would be better off if you were dead?” (never, some, a lot, and all the time); (b) “Since the last prompt, how often have you thought of killing yourself?” (never, some, a lot, and all the time); and (c) “Since the last prompt, how often have you self‐harmed?” (none, 1 time, 2–5 times, and 5+ times). However, there were too few positive responses to allow the analysis.

### 2.6. Social Feedback ERP Task

The Island Getaway task was used to measure brain responses to social feedback. Youth were told that they were playing a game with 11 coplayers in which they were traveling in the Hawaiian Islands. At each island, they voted whether they wanted each coplayer to continue with them to the next island, followed by feedback on how coplayers voted for them. Participants reviewed information about each coplayer (e.g., sex, location, and personal preferences) and entered their information, which they were told was reviewed by the coplayers. Participants completed six rounds of “voting,” during which they voted whether to “keep” or “kick out” each coplayer, followed by feedback as to whether that coplayer voted to accept (“keep”) or reject (“kick out”) them. After each round, participants were told that one of the coplayers had been kicked out, and after completing the sixth round, participants were informed that they had made it to the Big Island. Participants received 51 trials of social feedback: 25 acceptance and 25 rejection and one randomly selected trial. Coplayers were randomly assigned a voting pattern for each participant such that two coplayers rejected the participant on most rounds, two coplayers accepted the participant on most rounds, and the remaining coplayers were equally likely to accept or reject the participant. Following the task, participants completed a brief post‐task questionnaire assessing their task engagement. This task has been validated in early adolescent, late adolescent, and young adult samples [33, 35, 49–51]. We focus on the Wave 2 ERP data to align with the timing of the EARS data acquisition.

#### 2.6.1. EEG Acquisition and Processing

Continuous EEG was recorded using the BrainVision ActiChamp, a 32 channel active channel amplifier system (BrainVision LLC, Morrisville, NC, USA). Electrodes were mounted in an elastic cap using a subset of the International 10/20 System sites, with the ground electrode located at FPz. The electrooculogram (EOG), generated from blinks and eye movements, was recorded from five facial electrodes. The EEG was sampled at 500 Hz, and all signals were digitized on a computer. All data were re‐referenced to the average of Tp9 and Tp10 and band‐pass filtered from 0.1 to 30 Hz. The EEG was corrected for EOG artifacts [52] and physiological artifacts, which were removed using an automatic procedure with a maximum allowed voltage step of 50 µV within a 400 ms interval length, a maximum absolute difference between any two points of 175 µV, and a minimum allowed activity of 0.50 µV within a 100 ms interval length. The EEG was segmented into 1000 ms epochs, beginning 200 ms before and ending 800 ms after feedback onset. ERPs were averaged separately for acceptance and rejection feedback and baseline corrected to activity 200 ms prior to feedback.

Peak detection for the RewP (i.e., identifying the most positive peak of the acceptance–rejection difference waveform) was conducted independently for electrodes FZ and CZ within the 250−500 ms window following feedback. These peaks were then visually inspected and confirmed, or changed when appropriate, by two researchers aware of the general sample characteristics but blind to pertinent demographic details for individual participants, including group assignment. The majority of the peaks occurred within the 275–375 ms time window. The 100 ms area centered around the peak (RewP) was selected for analysis.

To expand upon prior findings with the RewP, and in line with previous work and recommendations [53], residual scores for the RewP response to acceptance, accounting for the response to rejection, were calculated, producing scores uncorrelated with the response to rejection. Likewise, residual scores for the response to rejection, accounting for the response to acceptance, were calculated. This produces scores that are correlated (*r* = 0.83), but not inverses of one another, and isolates the mean amplitude in the ERP unique to acceptance or rejection. A more negative residualized rejection response indicates a stronger response to negative social feedback, while a more positive residualized acceptance response indicates a stronger response to positive social feedback.

### 2.7. Statistical Plan

#### 2.7.1. Intensive Longitudinal Data

Goal #1: Do NRY experience greater interpersonal criticism/conflict, rejection sensitivity, depression, SI, and NA, as well as lower PA?

##### 2.7.1.1. Weekly/Monthly Assessment

These assessments allowed us to address our goals at the level of weekly/monthly experiences over one to 2 years. We asked whether NRY showed higher interpersonal criticism/conflict (parent or peer) and greater rejection sensitivity (burdensomeness or belonging) using multilevel linear models (MLMs), with FDR correction for the four models. Then using MLM models, we examined whether NRY showed greater depression and NA or reduced PA.

Hierarchical generalized linear models (HGLMs) with binomial distribution and logit link function were conducted to model the dichotomous variable (SI), following the same approach as the MLMs for the continuous outcomes. MLMs and HGLMs were performed in SAS version 9.4 using the PROC MIXED and GLIMMIX procedures (SAS Institute Inc., Cary, NC, 2016). These models account for multiple intensive longitudinal surveys per participant and allow for missing data and varying times between assessments. Intercept and age (centered within‐person) at the time the e‐survey was completed were modeled as both fixed and random effects with an unstructured covariance structure. A dichotomous wave variable identified whether e‐surveys were in the year after Wave 1 or 2, and sex was included as a between‐person covariate.

##### 2.7.1.2. Momentary Assessment

The momentary assessment allowed us to address our goals on a more granular level within a few days. We asked whether NRY showed greater momentary conflicts with peers or family, higher NA, or lower PA and connectedness, again using MLMs, with FDR correction for the five models.

Goal #2: Do NRY show stronger associations of interpersonal criticism/conflict and rejection sensitivity to depression, SI, NA, and PA?

##### 2.7.1.3. Weekly Assessment

We asked whether remission group status moderated the concurrent association of interpersonal criticism/conflict and rejection sensitivity to depression symptoms, SI, and broader affect (PA and NA) across weeks. In these MLM models, remission group and measures of either interpersonal criticism/conflict or rejection sensitivity, as well as the interaction between the group and the other measure, were used to model either depression, NA, or PA scores at each e‐survey in the year after Waves 1 and 2. FDR correction was used across the four models for each outcome. Predictors were fixed effects in the models and were centered within‐person. For any significant interaction between remission group and another measure, we then report pairwise group comparisons of the interaction, with FDR correction across the three group comparisons.

##### 2.7.1.4. Momentary Assessment

The goal of these analyses was to understand whether remission group status (at Wave 2) impacts the concurrent association of interpersonal criticism/conflict (family or peer) to affect (PA and NA) or connectedness on a more granular level within days. We again used MLMs: separate models for the three dependent variables (momentary PA, NA, and connectedness) and for the two independent variables (momentary conflict with peers and family). Interactions between the independent variables and remission groups were included in these models to test moderation. The MLMs included a random intercept, assumed an unstructured covariance structure, covaried for time of survey completion, and nested days within participants. We again used FDR to correct for multiple comparisons, with one family being the six main effects (momentary parent or peer conflict with NA, connectedness, or PA across groups) and the other family the six interactions with remission group for each type of analysis. For any significant interaction between remission group and either momentary parent or peer conflict, we then report pairwise group comparisons of the interaction, with FDR correction across the three group comparisons.

Goal #3: What is the direction of the associations between family and peer criticism/conflict and depression and affective disturbances, particularly among NRY?

##### 2.7.1.5. Weekly Assessment

We examined temporally lagged associations over weeks with family and peer criticism/conflict using the same MLM/HGLM analysis and FDR correction approach as for the concurrent associations, asking whether family or peer criticism/conflict at week *t* predicted depression, SI, NA, or PA at week *t + 1* but controlling for week *t* levels of the outcome variable. We then asked whether depression, SI, NA, or PA at week *t* predicted family or peer criticism/conflict at week *t + 1*, again controlling for family or peer criticism/conflict at week *t*. For each of these analyses, we assessed whether the association was stronger in non‐remitted (i.e., moderation). For these analyses, we only included youth who completed the e‐surveys weekly (*n* = 137), excluding those who only did monthly surveys.

##### 2.7.1.6. Momentary Assessment

We examined temporally lagged associations within days (i.e., predictors were from the survey prior to the outcomes, using data from the same day only), using the same MLM analysis and FDR correction approach as for the concurrent associations. Specifically, we asked whether (a) momentary family or peer conflicts at prompt *t* predicted momentary NA, connectedness, or PA at prompt *t + 1*, controlling for those variables at prompt *t*; or (b) momentary NA, connectedness, or PA at prompt *t* predicted momentary family or peer conflicts at prompt *t + 1*, controlling for those variables at prompt *t*.

Goal #4: Do NRY show blunted neural responses to positive social feedback and stronger neural responses to negative peer feedback?

##### 2.7.1.7. Social Rejection ERP Analysis

General linear regression models were conducted with Wave 2 ERP variables as the dependent variables and remission group at Wave 2 as the independent variable covarying for sex at birth and age at ERP. The primary ERP variables were peak amplitude for the RewP at FZ and CZ. When the omnibus *p*‐value survived FDR correction, pairwise group comparisons were reported, also with FDR correction. For any significant differences as a function of remission group, follow‐up analyses were run using the residualized acceptance and rejection peak amplitude.

## 3. Results

As shown in Table 1, the three remission groups (CCs, RY, and NRY) differed on age at final assessment, MDD core score at final assessment, and sex. NRY youth were significantly older and had greater depression severity than that of RY and CC subjects. The RY group had significantly fewer females than the NRY and CC groups. None of the other characteristics differed by remission group.

Goal #1: Do NRY experience greater interpersonal criticism/conflict, rejection sensitivity, depression, SI, and NA, as well as lower PA?

### 3.1. Weekly/Monthly Assessment Results

The means and standard deviations for each weekly survey measure as a function of group are shown in Table 2. As shown in Table 3, the remission groups differed significantly in reports of both parent and peer criticism/conflict as well as perceived burdensomeness and thwarted belongingness. The NRY youth reported higher levels of these four measures than the CC and RY groups, who did not differ significantly in parent criticism/conflict, peer criticism/conflict, perceived burdensomeness, and thwarted belongingness. For the analyses with depression, SI, and affect as dependent variables, the complete details (estimates, standard errors, *t*‐values, and *p*‐values) for all MLMs are shown in Tables S2–S5, including main effects of the remission group and predictors, as well as interactions. The NRY group reported significantly higher levels of depression (Table S2), SI (Table S3), and NA (Table S4), as well as lower PA (Table S5), compared to both the CC and RY groups.

**Table 2:** E‐survey (weekly) and EMA (momentary) mean and between‐person SD scores across e‐surveys and EMAs by remission group status.

E‐surveys (weekly)			
E‐survey variables	Community control	Remitted	Non‐remitted
	Mean (SD)	Mean (SD)	Mean (SD)
Parent criticism/conflict	19.90 (18.32)	17.60 (13.02)	27.50 (19.51)
Peer criticism/conflict	12.94 (13.50)	14.14 (12.58)	20.48 (17.44)
Perceived burdensomeness	4.28 (6.17)	8.54 (12.57)	16.78 (18.65)
Thwarted belongingness	11.74 (10.59)	14.56 (13.17)	23.83 (16.27)
Depression	0.11 (0.10)	0.17 (0.15)	0.40 (0.26)
Suicidal ideation	0.87% (2.87%)	3.60% (12.00%)	13.10% (21.39%)
Negative affect	24.14 (13.51)	25.92 (14.25)	35.54 (15.78)
Positive affect	70.13 (12.54)	67.22 (15.85)	60.90 (13.54)

**EMA surveys (momentary)**			

**EMA variables**	**Community control**	**Remitted**	**Non-remitted**
	**Mean (SD)**	**Mean (SD)**	**Mean (SD)**

Family conflicts	4.77 (6.01)	2.97 (4.53)	7.41 (7.76)
Friend conflicts	0.62 (1.05)	2.48 (4.48)	6.24 (8.22)
Negative affect	12.62 (14.94)	11.41 (11.77)	23.60 (14.29)
Connectedness	86.07 (18.34)	87.43 (12.73)	67.38 (17.30)
Positive affect	69.35 (20.37)	70.25 (18.04)	51.47 (20.82)

**Table 3:** Multilevel models of e‐survey (weekly) and EARS (momentary) variables by final assessment wave remission group status.

Dependent variable	Omnibus test: remission group		Community control vs. non‐remitted		Remitted vs. non‐remitted		Community control vs. remitted	
	*F*	*q*‐Value	*t*	*q*‐Value	*t*	*q*‐Value	*t*	*q*‐Value
E‐survey (weekly) variables (*N* = 159)								
Parent criticism/conflict	4.91	0.0170	−2.25	0.0384	−3.03	0.0087	0.41	0.6803
Peer criticism/conflict	3.71	0.0427	−2.40	0.0308	−2.34	0.0308	−0.40	0.6885
Perceived burdensomeness	10.10	<0.0001	−4.40	<0.0001	−3.06	0.0039	−1.89	0.0602
Thwarted belongingness	11.89	<0.0001	−4.67	<0.0001	−3.60	0.0006	−1.69	0.0935
EARS (momentary) variables (*N* = 53)								
Negative affect	4.91	0.0192	2.21	0.0323	−2.98	0.0060	0.17	0.8621
Connectedness	10.24	0.0010	−3.21	0.0096	4.29	0.0004	−0.23	0.8621
Positive affect	6.16	0.0103	−2.48	0.0225	3.33	0.0032	−0.18	0.8621
Friend conflicts	3.64	0.0435	2.55	0.0225	−1.95	0.0572	−1.00	0.8621
Family conflicts	2.04	0.1431	na	na	na	na	na	na

*Note: q*‐value is the FDR adjusted *p*‐value. “na” indicates not applicable since the omnibus test was not significant.

### 3.2. Momentary Assessment Results

As shown in Table 3, youth in the NRY group reported significantly greater NA and lower connectedness and PA than both the RY and CC groups. The NRY group also reported more frequent friend conflicts than the CC group, with the same trend compared to the RY group. There were no overall group differences in the reports of family conflicts.

Goal #2: Do NRY show stronger relations of interpersonal criticism/conflict and rejection sensitivity to depression, SI, and affective disturbances?

### 3.3. Weekly Assessment Results

#### 3.3.1. Main Effects of Predictors Across Groups

As shown in Table S2, each of the four predictors showed a main‐effect association with depression. Greater parent and peer criticism/conflict, perceived burdensomeness, and thwarted belongingness were associated with greater depression. All predictors were associated with a greater SI (Table S3). Each predictor again showed a significant main effect for NA (Table S4), consistent with the pattern observed for depression. The opposite pattern was found for PA (Table S5).

#### 3.3.2. Remission Group Differences in the Association Between Predictors and Outcomes

**Figure 1:**
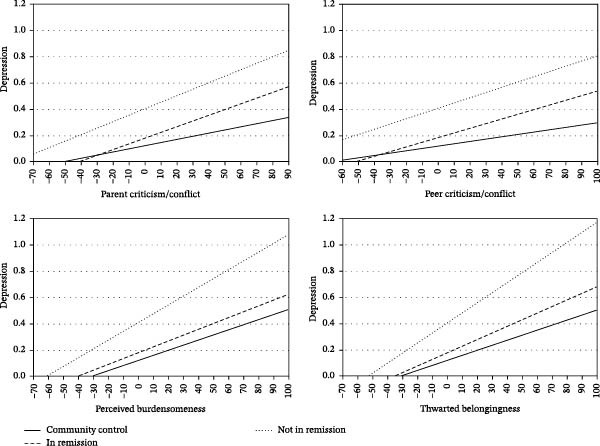
Predictors of depression that significantly interact with remission group in e‐surveys. Graphs plotting the association between predictors and depression as a function of remission group.

Table 4 focuses on the interaction of remission group and each predictor in the association to depression, SI, NA, and PA (full details in Tables S2–S5). Remission group significantly interacted with each of the four predictors to predict depression. The pairwise group comparisons indicated that the association between each predictor and depression differed significantly between the CC and NRY groups. As shown in Figure 1, for predictors (parent and peer criticism/conflict, burdensomeness, and thwarted belongingness), there is a significantly stronger positive association to depression in the NRY group compared to the CC and RY group (other than parent/peer criticism/conflict for RY) and a significantly stronger positive association in the CC compared to the RY group (other than perceived burdensomeness). Thus, across all predictors, there seemed to be the strongest association with depression in the NRY group as compared to that in the other groups. We did not find any significant interactions with remission group in predicting SI (Table 4).

**Table 4:** Multilevel models of e‐survey (weekly) depression, positive affect, negative affect, and suicidal ideation by the interaction between e‐survey risk factors and final assessment wave remission group status (*N* = 159).

Predictor	Omnibus test: predictor X remission group		Predictor X community control vs. non‐Remitted		Predictor X remitted vs. non‐remitted		Predictor X community control vs. remitted	
	*F*	*q*‐Value	*t*	*q*‐Value	*t*	*q*‐Value	*t*	*q*‐Value
DV = depression								
Parent criticism/conflict	11.21	<0.0001	−4.70	<0.0001	−1.84	0.0654	−3.58	0.0005
Peer criticism/conflict	8.17	0.0004	−4.02	<0.0001	−1.58	0.1138	−3.19	0.0021
Perceived burdensomeness	32.26	<0.0001	−4.12	<0.0001	−7.62	<0.0001	−0.78	0.4359
Thwarted belongingness	53.44	<0.0001	−7.48	<0.0001	−9.17	<0.0001	−2.35	0.0189
DV = suicidal ideation								
Parent criticism/conflict	0.48	0.6215	na	na	na	na	na	na
Peer criticism/conflict	2.46	0.2277	na	na	na	na	na	na
Perceived burdensomeness	1.19	0.4846	na	na	na	na	na	na
Thwarted belongingness	2.14	0.2348	na	na	na	na	na	na
DV = negative affect								
Parent criticism/conflict	11.09	<0.0001	3.71	0.0003	3.79	0.0003	1.49	0.1351
Peer criticism/conflict	11.76	<0.0001	3.99	<0.0001	3.77	0.0003	1.98	0.0475
Perceived burdensomeness	3.39	0.0339	2.02	0.0654	−1.17	0.2406	2.53	0.0348
Thwarted belongingness	4.30	0.0155	0.46	0.6449	−2.60	0.0285	1.97	0.0732
DV = positive affect								
Parent criticism/conflict	1.50	0.2559	na	na	na	na	na	na
Peer criticism/conflict	0.32	0.7252	na	na	na	na	na	na
Perceived burdensomeness	1.54	0.2559	na	na	na	na	na	na
Thwarted belongingness	12.70	<0.0001	1.53	0.1782	5.04	<0.0001	−1.35	0.1782

*Note: q*‐value is the FDR adjusted *p*‐value. “na” indicates not applicable since the omnibus test was not significant.

**Figure 2:**
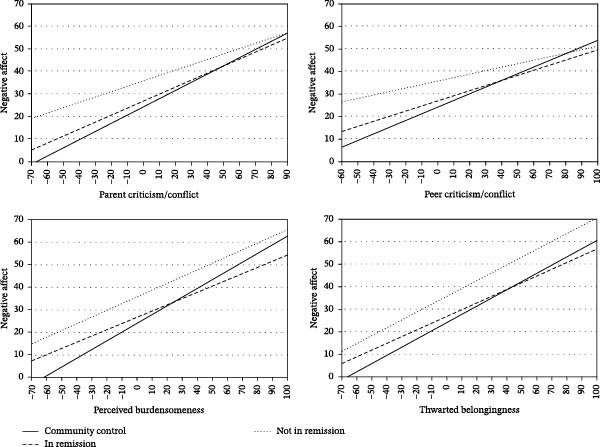
Predictors of negative affect that significantly interact with remission group in e‐surveys. Graphs plotting the association between predictors and depression as a function of remission group.

There were also significant group interactions with each of the predictors for NA (Table 4), but of a different pattern than that found for depression. The majority of interactions did not indicate stronger associations within the NRY group (Figure 2). Instead, peer and parent criticism/conflict were significantly more strongly positively associated with NA affect in the CC and RY groups than in the NRY group. The negative associations between perceived burdensomeness and thwarted belongingness and NA were significantly stronger in the CC and NRY groups than in the RY group. Thus, these results suggest that broader NA is not more closely tied to peer/family criticism/conflict or rejection sensitivity in the NRY group, unlike depression. There was only one significant group‐by‐predictor interaction for PA: a significantly stronger negative association between thwarted belongingness and PA in the NRY versus RY group, but neither differed from CC (Table 4, Figure S2).

### 3.4. Momentary Assessment Results

#### 3.4.1. Main Effects of Predictors Across Groups

As shown in Table S6, there were main effects of youth reporting friend and family conflicts, with the occurrence of conflicts associated with greater NA, less connectedness, and lower PA.

### 3.5. Remission Group Differences in the Association Between Predictors and Outcomes

As shown in Table S6, there was one significant interaction of remission group with family conflicts in predicting feelings of connectedness. As shown in Figure S3, there was a stronger relationship between the occurrence of family conflicts and reduced feelings of connectedness in the NRY group versus the RY and CC groups.

Goal #3: What is the direction of the associations between family and peer criticism/conflict and depression and affective disturbances, particularly among NRY?

### 3.6. Weekly Assessment Results

We did not see any lagged associations between parent/peer criticism/conflict and depression with SI, NA, or PA that differed by remission group in the weekly surveys in either direction. However, as shown in Tables S7 and S8, we did observe evidence of bidirectional associations between parent/peer criticism/conflict across remission groups. Specifically, as shown in Table S7, parent criticism/conflict at week *t* predicted greater SI, greater NA, and lower PA at week *t + 1*, accounting for values of these outcome variables at week *t* after FDR correction. Furthermore, greater peer criticism/conflict at week *t* predicted greater NA at week *t + 1*, accounting for NA at week *t*. However, as shown in Table S8, we also saw the reverse association, with depression, NA, and PA at week *t* predicting greater parent and peer criticism/conflict at *week t + 1*, accounting for parent and peer criticism/conflict at week *t*.

### 3.7. Momentary Assessment Results

**Figure 3:**
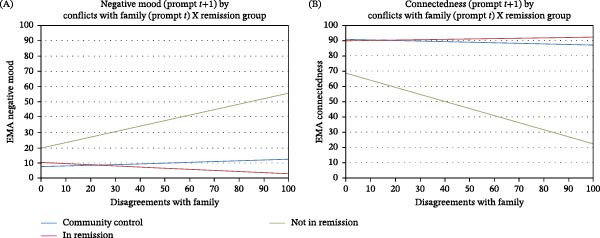
Association of family conflicts and negative affect and connectedness in time‐lagged ecological momentary assessment analyses. (A) Associations between family conflicts and negative affect as a function of remission group. (B) Associations between family conflicts and connectedness as a function of remission group status.

As shown in Table 5 and Table S9, some of the associations between friend/family conflicts to affect and connectedness differed significantly across groups in the time‐lagged analyses. As shown in Figure 3A, family conflicts at prompt *t* were significantly more strongly positively related to NA at prompt *t + 1* in the NRY versus RY groups, with a similar but nonsignificant pattern for the comparison of NRY versus CC. In addition, there was a significantly stronger negative association between family conflicts at prompt *t* and lower connectedness at prompt *t + 1* in the NRY group compared to the RY group (Figure 3B, again with a similar but nonsignificant pattern for the comparison of NRY versus CC). Friend conflicts at prompt *t* also predicted NA at prompt *t + 1*, but this association did not differ by remission group. Thus, while relations did not differ in the concurrent analyses, family conflicts were significantly more strongly related to NA and connectedness at a subsequent prompt in the NRY group, suggesting potentially longer‐lasting effects of interpersonal conflicts with family on NA in the NRY group. There were no time‐lagged associations in the other direction that passed FDR correction, either across groups or that differed by remission group (Table S10).

**Table 5:** Multilevel models of EMA positive affect, negative affect, and connectedness by Wave 2 remission group status and conflicts with friends and family at the prior prompt from the same day.

Dependent variable	Omnibus test		Remitted vs. community control		Non‐Remitted vs. community control		Remitted vs. non‐remitted	
	*F*	*q*‐Value	*t*	*q*‐Value	*t*	*q*‐Value	*t*	*q*‐Value
DV = negative affect								
M1: friend conflicts	12.43	0.0010	–					
M2: friend conflicts X remission group	2.74	0.1342	na	na	na	na	na	na
M1: family conflicts	14.38	0.0006	–					
M2: family conflicts X remission group	5.96	0.0135	−0.51	0.6108	1.31	0.2868	−3.42	0.0021
DV = connectedness								
M1: friend conflicts	0.57	0.5392	–					
M2: friend conflicts X remission group	1.72	0.2109	na	na	na	na	na	na
M1: family conflicts	16.34	<0.0001	–					
M2: family conflicts X remission group	5.54	0.0135	0.22	0.8259	−1.51	0.1973	3.26	0.0039
DV = positive affect								
M1: friend conflicts	0.29	0.5896	–					
M2: friend conflicts X remission group	1.95	0.2109	na	na	na	na	na	na
M1: family conflicts	4.34	0.0573	–					
M2: family conflicts X remission group	1.57	0.2109	na	na	na	na	na	na

*Note:* Model 1 (M1) included only the main effect of the independent variable and Model 2 (M2) included the interaction between the independent variable and Wave 2 remission group. “na” means not applicable since the omnibus test was not significant.

### 3.8. ERP Results

Goal #4: Do NRY show blunted neural responses to positive social feedback and stronger neural responses to negative peer feedback?

**Figure 4:**
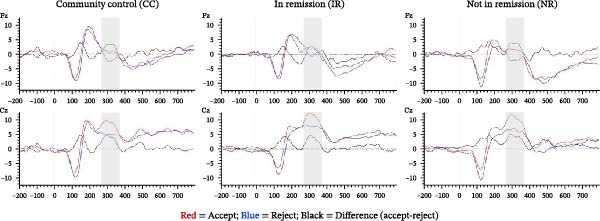
Grand average wave forms for each remission group for the reward positivity (RewP) at FZ and CZ. The shaded area of each group indicates the time period in which the majority of peaks were identified across participants.

As shown in Table 6A, there were significant remission group differences in the magnitude of the RewP to peer rejection versus acceptance at FZ, with a similar trend at CZ. Pairwise analyses revealed that the RewP was significantly larger in the NRY group compared to that in the RY group. The RewP was also numerically larger in the NRY group compared to the CC, but this difference was not statistically significant. As shown in Table 6B and Figure 4, this effect seemed to be related to a significantly more negative waveform to rejection in the NRY group compared to the RY group, with the same pattern compared to CC. There was also a significant group difference in the acceptance waveform, but none of the pairwise comparisons were significant.

**Table 6:** General linear regressions of Wave 2 ERP variables by Wave 2 remission group status covarying for sex at birth and age at ERP.

Dependent variable	Community control (*N* = 27)	Remitted (*N* = 44)	Non‐remitted (*N* = 26)	Omnibus test	Remitted vs. community control	Non‐remitted vs. community control	Remitted vs. non‐remitted
	Mean (SD)	Mean (SD)	Mean (SD)	*F* (*q*‐value)	*t* (*q*‐value)	*t* (*q*‐value)	*t* (*q*‐value)
(A) ERP variable							
RewP FZ	1.01 (5.81)	0.12 (6.38)	2.60 (5.19)	4.79 (0.0210)	−1.86 (0.0995)	1.11 (0.2681)	−3.02 (0.0102)
RewP CZ	2.38 (4.92)	1.89 (5.27)	3.67 (5.51)	2.86 (0.0624)	na	na	na
(B) Follow‐up analyses for significant FZ effect							
Rejection residual	0.15 (4.72)	0.58 (5.97)	−2.07 (5.12)	5.75 (0.0088)	1.47 (0.1447)	−1.81 (0.1098)	3.39 (0.0030)
Acceptance residual	0.29 (6.02)	−0.83 (5.68)	0.91 (4.94)	3.17 (0.0465)	−1.94 (0.0828)	0.33 (0.7410)	−2.24 (0.0828

*Note: q*‐value is the FDR adjusted *p*‐value. “na” indicates not applicable because the omnibus test was not significant.

## 4. Discussion

**Figure 5:**
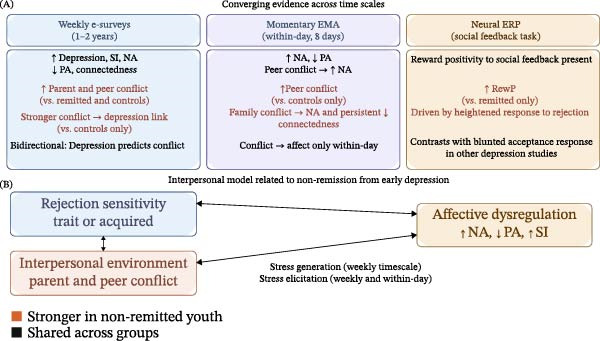
Converging evidence for interpersonal reactivity as a correlate of non‐remission from early‐onset depression across time scales and assessment modalities. (A) Findings from three complementary assessment approaches are summarized. Text in coral/red indicates findings that were stronger in or specific to non‐remitted youth relative to remitted youth and/or community controls; black text indicates findings shared across groups. (B) Interpersonal model related to non‐remission from early depression.

Across multiple time scales and converging methods, we found evidence that youth with a history of PO‐MDD whose depression was not remitted at adolescence experienced greater parent and peer criticism/conflict, greater rejection sensitivity, and decreased connectedness. As would be expected, they had greater depression, SI, and NA, and lower PA. Further, on a weekly basis over a period of 1–2 years, NRY exhibited a stronger association between experiencing interpersonal conflict and depression compared to CCs, with a similar trend compared to RY. On a within‐day timescale, in momentary assessments, NRY showed a longer‐lasting association between family conflicts and both NA and connectedness than RY (i.e., an association that persisted beyond the current prompt to the next prompt). In addition, there was converging evidence from ERP measures of a stronger response to negative social feedback among NRY. Thus, across different time scales (weekly and momentary) and types of assessments (self‐report and neural responses), we see evidence that NRY experience a greater negative impact from interpersonal conflict or feedback. Taken together, and summarized in Figure 5, these findings suggest that many of the interpersonal factors associated with depression relapse and recurrence in older adolescents and adults are also related to remission status among youth with a history of PO‐MDD, suggesting that these are potentially key, currently under‐prioritized, interpersonal intervention targets earlier in childhood.

Not surprisingly, NRYh showed greater depression, SI, and NA, as well as lower PA and connectedness than RY and CCs. This was true for both intensive longitudinal assessment scales, including the longer time scale, measured by weekly e‐surveys over one to 2 years, and on the shorter time scale of momentary assessments over 1 week, measured via EMA.

Our data provide strong evidence that interpersonal factors/reactivity may be central to understanding remission from PO‐MDD in adolescent youth. First, over the longer time scale provided by the e‐survey data, NRY reported greater criticism/conflict with both parents and peers compared to RY and CCs. We also observed higher reports of peer conflicts in the momentary assessments across 8 days but not of family conflicts. The longer time scale of the weekly surveys may have provided more opportunities to capture instances of such family conflicts, which may not necessarily occur on a momentary basis. In addition, the weekly surveys asked specifically about parent criticism/conflict. In contrast, the momentary surveys inquired about family conflicts more broadly (which could include sibling conflicts). Thus, it is possible that NRY specifically experience more parent conflict rather than broader family criticism/conflict, which was better captured in the weekly surveys.

These data are consistent with the literature on parental expressed criticism [9, 10, 12, 14] and parent–child conflict [15] as a risk factor for depression onset and recurrence. They are also consistent with findings that peer victimization [19–21] and peer conflict [2, 3] are associated with depression. As noted in the introduction, there is also evidence for bidirectional effects at least on the scale of weeks, with depression in youth predicting greater levels of criticism [16, 17]. This pattern is consistent with the stress generation hypothesis of depression [54, 55], which suggests that the experience of depression can contribute to greater interpersonal criticism/conflict and stressors, both because of how the individual with depression responds to others and because other aspects of depression (e.g., lack of motivation, sleep difficulties, attentional difficulties) may lead to behaviors that elicit negative feedback or anger from others. In our weekly assessments, we saw evidence for a bidirectional association, with parent or peer criticism/conflict in 1 week predicting greater SI and NA, as well as lower PA, in the subsequent week. At the same time, greater depression, worse NA, and lower PA predicted greater parent or peer criticism/conflict in the subsequent week. Interestingly, on the shorter time scale captured by the EMA surveys (momentary), there was only evidence that family or friend conflicts predicted subsequent greater NA and lower connectedness, but not vice versa. These data are consistent with the possibility that stress generation operates at a somewhat longer time scale – over the course of a week or weeks, but that at the short‐time scale of within days, parent or peer conflict primarily operates by generating risk for subsequent NA and interpersonal disconnection. However, it is also possible that the cumulative effects of within‐day interpersonal risk are part of the pathway that leads to stress generation across longer time scales as ongoing NA may begin to elicit greater interpersonal conflict over time (Figure 5).

Our findings suggest that the association between family or peer criticism/conflict and depression may be stronger for youth who are particularly sensitive to negative social feedback or perceived rejection from others. Consistent with this interpretation and with prior work in adolescent depression [4, 26], NRY show increased rejection sensitivity compared to RY or CCs. First, NRY reported higher levels of burdensomeness and lower belongingness/connectedness, both at the weekly survey time scale and at the EMA time scale. Second, NRY had a stronger association between experiencing peer and family criticism/conflict and greater depression levels as compared to RY and CCs. Intriguingly, we only found this association for a depressed mood and not for NA. In fact, there was a weaker association between parent/peer criticism/conflict and NA in the NRY. Speculatively, this may point to some specificity in the emotional impact of rejection or criticism/conflict sensitivity on depression rather than a broader range of negative affective responses. Nonetheless, we saw a hint of an effect for NA in the EMA data. In the EMA data, peer conflicts were associated with greater NA and connectedness, and family conflicts were associated with lower PA concurrently for all youth. However, there was a stronger association between family conflicts and both NA and connectedness at the subsequent prompt in NRY. This may indicate a longer‐lasting impact of family conflicts on NRY. Our original hypothesis was that this type of rejection sensitivity may be a trait‐like characteristic that puts youth at risk for the negative impact of interpersonal conflict. However, we cannot rule out the possibility that such rejection sensitivity is the result of chronic interpersonal conflicts that engender greater sensitivity to such conflict. In either case, helping youth to become aware of such heightened reactivity and develop skills to cope with this reactivity earlier in childhood may be clinically useful and could subsequently improve both peer and family associations.

Our ERP data also provided some evidence consistent with greater rejection sensitivity among NRY from depression at adolescence, in alignment with both the weekly and momentary assessments (Figure 5). Specifically, we saw a larger RewP to social feedback in NRY compared to RY but not compared to CCs, though the trend was in the same direction. This latter comparison may have lower power due to the smaller CC versus the RY sample. This effect was primarily driven by a stronger response to rejection among NRY. This pattern contrasts with previous ERP studies of social feedback, which have been more likely to find blunted responses to social acceptance specifically in those experiencing depression rather than a stronger response to social rejection [32, 34, 35]. However, our findings are consistent with several fMRI studies that have found increased activity in regions such as the amygdala, anterior cingulate, and insula in response to social rejection in depressed groups [29, 31]. It is unclear what leads studies to find effects primarily as a blunted response to social acceptance versus a heightened response to social rejection in relation to depression in adolescents. One possibility is that this could reflect heterogeneity in symptom presentation in depression, with higher levels of anhedonia potentially associated with stronger effects for social acceptance but higher levels of rejection sensitivity or interpersonal stress associated with stronger effects for social rejection. For example, research has shown that depressed individuals with high levels of anhedonia show a blunted RewP to social gain versus neutral feedback [56]. Studies with larger samples that can dissociate associations between different depression symptoms and behavioral and neural measures of social feedback processing are needed to test such hypotheses.

This work had several strengths, including longitudinal data collected at multiple time scales (weekly and momentary) and complementary evidence from the neural data. However, these findings should be considered in the context of several limitations. First, we examined experiences in NRY who were experiencing higher levels of depression and NA at the time of assessment. We do not know from these data whether these youth have similar experiences when they are not depressed and, if so, whether this predicts the likelihood of relapse, important questions to be tested in future studies. We also do not know whether this interpersonal sensitivity is observed at younger ages or emerges across middle childhood, potentially in response to increases in experienced conflict/criticism. Second, we defined remission as a function of which youth were experiencing a major depressive episode at the time of their last assessment, labeling youth with a history of PO‐MDD who were not experiencing a depressive episode as remitted. It is possible that some of those youth with a history of PO‐MDD might have experienced another major depressive episode prior to their last assessment or will have in the future. Notably, we observed many differences between youth defined as remitted and those not, providing construct validity for our definition [57]. Third, our sample size and number of surveys for the EMA analyses were smaller than for the e‐survey data, resulting in less power to detect significant interactions with the remission group and potentially for the time‐lagged models. Fourth, we did not have samples of youth with a history of another form of psychopathology other than PO‐MDD or samples of youth with adolescent depression who never had PO‐MDD, so we do not know the specificity of these interpersonal and rejection sensitivity challenges experienced by NRY. However, findings on associations between family and peer criticism/conflict and depression, rather than broader PA or NA, which include anger and anxiety, are suggestive of some depression specificity and extend prior findings on interpersonal risk factors in adolescent depression onset to a sample with PO‐MDD.

## 5. Conclusions

In summary, the current study provided evidence that youth with a history of PO‐MDD who were not remitted at adolescence experienced greater parent and peer criticism/conflict and were more reactive to such criticism/conflict, consistent with the literature in older adolescents and adults. These data provided converging evidence from both self‐reports at multiple time scales and neural measures, consistent with the hypothesis that interpersonal stress may put youth at risk for ongoing chronic depression. Although there was evidence of interpersonal stress generation, such that greater depression and NA, as well as lower NA, predicted subsequent family and peer/friend criticism/conflict, the strength of these associations was similar in the NRY. Together, these data point to the need to address interpersonal functioning and interpersonal reactivity as targets for early intervention that might help reduce relapse among youth who experience early‐onset depression.

## Funding

This study was supported by the National Institute of Mental Health (Grants R01MH117436, K01MH127412, K23MH125023, and K23MH118426).

## Conflicts of Interest

Deanna M. Barch consults for Jansen & Jansen and Boehringer Ingelheim. The other authors have no conflicts of interest.

## Supporting Information

Additional supporting information can be found online in the Supporting Information section.

## Supporting information


**Supporting Information** Supplemental Methods. Table S1: Subject characteristics in those included in versus excluded from analyses. Table S2: Details of multilevel models of e‐survey depression with interaction between e‐survey risk factors and final assessment wave remission group (NN = 159). Table S3: Details of multilevel models of e‐survey suicidal ideation with interaction between e‐survey risk factors and final assessment wave remission group (NN = 159). Table S4: Details of multilevel models of e‐survey negative affect with interaction between e‐survey risk factors and final assessment wave remission group (NN = 159). Table S5: Details of multilevel models of e‐survey positive affect with interaction between e‐survey risk factors and final assessment wave remission group (NN = 159). Table S6: Details of multilevel models of EARS positive affect, negative affect, and connectedness by Wave 2 remission group and conflicts with friends and family at the same prompt (NN = 53). Table S7: Details of multilevel models of e‐survey depression, suicidal ideation, negative affect, and positive affect with parent/peer conflict at prior week (NN = 137). Table S8: Details of multilevel models of e‐survey parent/peer conflict with depression, suicidal ideation, negative affect, and positive affect at prior week (NN = 137). Table S9: Details of multilevel models of EARS negative affect, connectedness, and positive affect by Wave 2 remission group and conflicts with friends and family at the prior prompt from the same day (NN = 53). Table S10: Details of multilevel models of EARS friend conflicts and family conflicts by Wave 2 remission group and negative affect, positive affect, and connectedness at the prior prompt from the same day (NN = 53). Figure S1: Timeline of assessments and sample sizes. Figure S2: Thwarted belongingness X remission group positive affect in concurrent model. Figure S3: Family conflicts X remission group connectedness in concurrent model.

## Data Availability

The data that support the findings of this study are available upon request from the corresponding author. The data are not publicly available due to privacy or ethical restrictions.
