# Supplementary material for: Reactivity to Interpersonal Conflict: A Correlate of Non‐Remission in Childhood Depression—Evidence Across Modalities and Time Scales
Source: Depress Anxiety. 2026 Jul 28;2026:5803578. doi: 10.1155/da/5803578 (PMC13409036; doi:10.1155/da/5803578)

**Supplemental Methods**

**E-Survey Completion Rates**

In the Wave 1 weekly surveys, youth included in this analysis completed 4,144 of 5,397 e-surveys (completion rate by participant: *M* = 79.1%, *SD* = 25.8%), and in the Wave 2 weekly surveys, youth completed 3,164 of 4,693 e-surveys (completion rate by participant: *M* = 70.1%, *SD* = 28.1%).

**Timing of EMA prompts, EMA methods, and EMA Payment**

On weekdays, youth were prompted to complete EMA surveys three times, with randomized timing of prompts between the following periods: (a) 6:00 AM and 8:00 AM; (b) 4:00 PM and 6:00 PM; and (c) 6:00 PM and 8:00 PM. On weekends, youth were prompted to complete EMA surveys four times, with randomized timing of prompts within each period: (a) 10:00 AM and 12:00 PM; (b) 12:00 PM and 2:00 PM; (c) 4:00 PM and 6:00 PM; and (d) 6:00 PM and 8:00 PM. For the remaining 30 days, youth were prompted to complete only a daily survey—these data are not included because only affect was assessed.

During the EMA period, the app provided feedback to youth about their survey completion and bonuses earned, and utilized gamification features (e.g., graphics and trophies) to reinforce adherence. Smartphone data was continuously uploaded to a cloud server and monitored so that youth who missed consecutive surveys or uninstalled the app were contacted and offered assistance. Youth received up to $149.50 (USD) for EMA and Daily Affect Survey completion, including bonuses for completing all surveys per day and 80% overall adherence.

**Supplemental Table S1. Subject Characteristics in those Included in vs. Excluded from Analyses**

|  | **Included in Analyses**  **(N=161)** | | | **Excluded from Analyses**  **(N=207)** | | | **Included vs.**  **Excluded** | |
| --- | --- | --- | --- | --- | --- | --- | --- | --- |
| **Subject Characteristics** | **N** | **Mean** | **SD** | **N** | **Mean** | **SD** | **t** | **p** |
| Baseline age | 161 | 5.34 | 1.01 | 207 | 4.98 | 1.05 | 3.31 | 0.0010 |
| Baseline income-to-needs ratio | 161 | 3.05 | 1.25 | 168 | 2.90 | 1.35 | 1.08 | 0.2816 |
| N therapy sessions | 121 | 17.49 | 5.92 | 165 | 7.76 | 9.36 | 10.04 | <0.0001 |
| Baseline MDD core score | 135 | 5.19 | 2.14 | 177 | 4.27 | 2.19 | 3.72 | 0.0002 |
|  | **N** | **%** | **n** | **N** | **%** | **n** | **χ^2^** | **p** |
| Female sex | 161 | 36.0 | 58 | 207 | 38.2 | 79 | 0.18 | 0.6736 |
| Hispanic ethnicity | 161 | 7.5 | 12 | 207 | 14.0 | 29 | 3.93 | 0.0474 |
| Race | 161 |  |  | 207 |  |  | F.E. | 0.0223 |
| White |  | 81.4 | 131 |  | 68.1 | 141 |  |  |
| Black |  | 9.3 | 15 |  | 16.9 | 35 |  |  |
| Asian |  | 1.9 | 3 |  | 1.5 | 3 |  |  |
| Multiracial |  | 7.5 | 12 |  | 13.5 | 28 |  |  |

F.E. = Fisher’s Exact Test

*Community control subjects not included

**Supplemental Table S2. Details of Multilevel Models of E-Survey Depression with Interaction between E-Survey Risk Factors and Final Assessment Wave Remission Group (N=159)**

|  | **DV = Depression** | | | |
| --- | --- | --- | --- | --- |
| **IV = Parent Criticism/Conflict** | **Estimate** | **SE** | **t** | **p** |
| Intercept | 0.403 | 0.030 | 13.45 | <0.0001 |
| Age | 0.039 | 0.017 | 2.25 | 0.0257 |
| Wave 1 vs. Wave 2 | -0.033 | 0.005 | -6.49 | <0.0001 |
| Female sex | 0.042 | 0.030 | 1.37 | 0.1722 |
| Parent criticism/conflict | 0.005 | 0.000 | 23.16 | <0.0001 |
| CC vs. NRY | -0.284 | 0.039 | -7.23 | <0.0001 |
| RY vs. NRY | -0.225 | 0.034 | -6.63 | <0.0001 |
| Parent criticism/conflict X CC vs. NRY | -0.003 | 0.001 | -4.70 | <0.0001 |
| Parent criticism/conflict X RY vs. NRY | -0.001 | 0.000 | -1.84 | 0.0654 |
| **IV = Peer Criticism/Conflict** | **Estimate** | **SE** | **t** | **p** |
| Intercept | 0.396 | 0.030 | 13.19 | <0.0001 |
| Age | 0.026 | 0.018 | 1.44 | 0.1514 |
| Wave 1 vs. Wave 2 | -0.023 | 0.005 | -4.46 | <0.0001 |
| Female sex | 0.043 | 0.031 | 1.42 | 0.1580 |
| Peer criticism/conflict | 0.004 | 0.000 | 18.36 | <0.0001 |
| CC vs. NRY | -0.287 | 0.039 | -7.28 | <0.0001 |
| RY vs. NRY | -0.223 | 0.034 | -6.55 | <0.0001 |
| Peer criticism/conflict X CC vs. NRY | -0.002 | 0.001 | -4.02 | <0.0001 |
| Peer criticism/conflict X RY vs. NRY | -0.000 | 0.000 | -1.58 | 0.1138 |
| **IV = Perceived Burdensomeness** | **Estimate** | **SE** | **t** | **p** |
| Intercept | 0.403 | 0.030 | 13.56 | <0.0001 |
| Age | 0.013 | 0.016 | 0.81 | 0.4175 |
| Wave 1 vs. Wave 2 | -0.028 | 0.005 | -5.70 | <0.0001 |
| Female sex | 0.037 | 0.030 | 1.23 | 0.2217 |
| Perceived burdensomeness | 0.007 | 0.000 | 31.90 | <0.0001 |
| CC vs. NRY | -0.289 | 0.039 | -7.41 | <0.0001 |
| RY vs. NRY | -0.227 | 0.034 | -6.75 | <0.0001 |
| Perceived burdensomeness X CC vs. NRY | -0.003 | 0.001 | -4.12 | <0.0001 |
| Perceived burdensomeness X RY vs. NRY | -0.002 | 0.000 | -7.62 | <0.0001 |
| **IV = Thwarted Belongingness** | **Estimate** | **SE** | **t** | **p** |
| Intercept | 0.395 | 0.030 | 13.25 | <0.0001 |
| Age | 0.023 | 0.015 | 1.50 | 0.1359 |
| Wave 1 vs. Wave 2 | -0.018 | 0.005 | -3.82 | 0.0001 |
| Female sex | 0.036 | 0.030 | 1.19 | 0.2363 |
| Thwarted belongingness | 0.008 | 0.000 | 34.91 | <0.0001 |
| CC vs. NRY | -0.285 | 0.039 | -7.27 | <0.0001 |
| RY vs. NRY | -0.226 | 0.034 | -6.66 | <0.0001 |
| Thwarted belongingness X CC vs. NRY | -0.004 | 0.001 | -7.48 | <0.0001 |
| Thwarted belongingness X RY vs. NRY | -0.003 | 0.000 | -9.17 | <0.0001 |

CC=Community control; RY=Remitted youth; NRY=Non-remitted youth; shading indicates passed FDR correction for interactions with remission group

**Supplemental Table S3. Details of Multilevel Models of E-Survey Suicidal Ideation with Interaction between E-Survey Risk Factors and Final Assessment Wave Remission Group (N=159)**

|  | **DV = Suicidal Ideation** | | | |
| --- | --- | --- | --- | --- |
| **IV = Parent Criticism/Conflict** | **Estimate** | **SE** | **t** | **p** |
| Intercept | -3.501 | 0.362 | -9.67 | <0.0001 |
| Age | -0.216 | 0.327 | -0.66 | 0.5093 |
| Wave 1 vs. Wave 2 | -0.024 | 0.141 | -0.17 | 0.8670 |
| Female sex | 1.238 | 0.397 | 3.12 | 0.0018 |
| Parent criticism/conflict | 0.022 | 0.004 | 5.54 | <0.0001 |
| CC vs. NRY | -3.313 | 0.664 | -4.99 | <0.0001 |
| RY vs. NRY | -1.705 | 0.413 | -4.13 | <0.0001 |
| Parent criticism/conflict X CC vs. NRY | -0.017 | 0.028 | -0.62 | 0.5358 |
| Parent criticism/conflict X RY vs. NRY | 0.005 | 0.007 | 0.70 | 0.4828 |
| **IV = Peer Criticism/Conflict** | **Estimate** | **SE** | **t** | **p** |
| Intercept | -3.630 | 0.368 | -9.87 | <0.0001 |
| Age | -0.258 | 0.346 | -0.75 | 0.4564 |
| Wave 1 vs. Wave 2 | 0.093 | 0.144 | 0.65 | 0.5166 |
| Female sex | 1.313 | 0.401 | 3.27 | 0.0011 |
| Peer criticism/conflict | 0.023 | 0.004 | 6.13 | <0.0001 |
| CC vs. NRY | -3.674 | 0.740 | -4.97 | <0.0001 |
| RY vs. NRY | -1.677 | 0.416 | -4.03 | <0.0001 |
| Peer criticism/conflict X CC vs. NRY | -0.083 | 0.037 | -2.22 | 0.0266 |
| Peer criticism/conflict X RY vs. NRY | -0.001 | 0.006 | -0.09 | 0.9286 |
| **IV = Perceived Burdensomeness** | **Estimate** | **SE** | **t** | **p** |
| Intercept | -3.816 | 0.380 | -10.05 | <0.0001 |
| Age | -0.475 | 0.308 | -1.54 | 0.1251 |
| Wave 1 vs. Wave 2 | 0.141 | 0.151 | 0.93 | 0.3525 |
| Female sex | 1.316 | 0.411 | 3.20 | 0.0014 |
| Perceived burdensomeness | 0.045 | 0.004 | 11.82 | <0.0001 |
| CC vs. NRY | -3.259 | 0.690 | -4.72 | <0.0001 |
| RY vs. NRY | -1.605 | 0.428 | -3.75 | 0.0002 |
| Perceived burdensomeness X CC vs. NRY | -0.041 | 0.033 | -1.25 | 0.2113 |
| Perceived burdensomeness X RY vs. NRY | -0.006 | 0.006 | -1.00 | 0.3151 |
| **IV = Thwarted Belongingness** | **Estimate** | **SE** | **t** | **p** |
| Intercept | -3.767 | 0.368 | -10.24 | <0.0001 |
| Age | -0.250 | 0.353 | -0.71 | 0.4786 |
| Wave 1 vs. Wave 2 | 0.202 | 0.147 | 1.37 | 0.1702 |
| Female sex | 1.261 | 0.396 | 3.19 | 0.0014 |
| Thwarted belongingness | 0.036 | 0.004 | 8.76 | <0.0001 |
| CC vs. NRY | -3.267 | 0.688 | -4.75 | <0.0001 |
| RY vs. NRY | -1.685 | 0.413 | -4.08 | <0.0001 |
| Thwarted belongingness X CC vs. NRY | -0.082 | 0.040 | -2.07 | 0.0385 |
| Thwarted belongingness X RY vs. NRY | -0.001 | 0.006 | -0.10 | 0.9222 |

CC=Community control; RY=Remitted youth; NRY=Non-remitted youth; shading indicates passed FDR correction for interactions with remission group

**Supplemental Table S4. Details of Multilevel Models of E-Survey Negative Affect with Interaction between E-Survey Risk Factors and Final Assessment Wave Remission Group (N=159)**

|  | **DV = Negative Affect** | | | |
| --- | --- | --- | --- | --- |
| **IV = Parent Criticism/Conflict** | **Estimate** | **SE** | **t** | **p** |
| Intercept | 33.525 | 2.390 | 14.02 | <0.0001 |
| Age | -1.991 | 1.182 | -1.68 | 0.0943 |
| Wave 1 vs. Wave 2 | 0.321 | 0.317 | 1.01 | 0.3114 |
| Female sex | 4.159 | 2.435 | 1.71 | 0.0896 |
| Parent criticism/conflict | 0.238 | 0.013 | 18.04 | <0.0001 |
| CC vs. NRY | -11.390 | 3.135 | -3.63 | 0.0004 |
| RY vs. NRY | -8.991 | 2.725 | -3.30 | 0.0012 |
| Parent criticism/conflict X CC vs. NRY | 0.123 | 0.033 | 3.71 | 0.0002 |
| Parent criticism/conflict X RY vs. NRY | 0.073 | 0.019 | 3.79 | 0.0002 |
| **IV = Peer Criticism/Conflict** | **Estimate** | **SE** | **t** | **p** |
| Intercept | 33.111 | 2.400 | 13.80 | <0.0001 |
| Age | -2.761 | 1.194 | -2.31 | 0.0222 |
| Wave 1 vs. Wave 2 | 0.810 | 0.328 | 2.47 | 0.0134 |
| Female sex | 4.353 | 2.447 | 1.78 | 0.0772 |
| Peer criticism/conflict | 0.156 | 0.014 | 11.40 | <0.0001 |
| CC vs. NRY | -11.611 | 3.150 | -3.69 | 0.0003 |
| RY vs. NRY | -8.789 | 2.738 | -3.21 | 0.0016 |
| Peer criticism/conflict X CC vs. NRY | 0.140 | 0.035 | 3.99 | <0.0001 |
| Peer criticism/conflict X RY vs. NRY | 0.071 | 0.019 | 3.77 | 0.0002 |
| **IV = Perceived Burdensomeness** | **Estimate** | **SE** | **t** | **p** |
| Intercept | 33.636 | 2.380 | 14.13 | <0.0001 |
| Age | -3.490 | 1.157 | -3.02 | 0.0030 |
| Wave 1 vs. Wave 2 | 0.425 | 0.315 | 1.35 | 0.1780 |
| Female sex | 3.748 | 2.425 | 1.55 | 0.1242 |
| Perceived burdensomeness | 0.299 | 0.013 | 22.36 | <0.0001 |
| CC vs. NRY | -11.651 | 3.123 | -3.73 | 0.0003 |
| RY vs. NRY | -8.972 | 2.713 | -3.31 | 0.0012 |
| Perceived burdensomeness X CC vs. NRY | 0.088 | 0.044 | 2.02 | 0.0436 |
| Perceived burdensomeness X RY vs. NRY | -0.022 | 0.019 | -1.17 | 0.2406 |
| **IV = Thwarted Belongingness** | **Estimate** | **SE** | **t** | **p** |
| Intercept | 33.148 | 2.401 | 13.81 | <0.0001 |
| Age | -2.968 | 1.116 | -2.66 | 0.0087 |
| Wave 1 vs. Wave 2 | 0.998 | 0.310 | 3.22 | 0.0013 |
| Female sex | 3.970 | 2.453 | 1.62 | 0.1075 |
| Thwarted belongingness | 0.350 | 0.014 | 24.76 | <0.0001 |
| CC vs. NRY | -11.600 | 3.155 | -3.68 | 0.0003 |
| RY vs. NRY | -8.930 | 2.746 | -3.25 | 0.0014 |
| Thwarted belongingness X CC vs. NRY | 0.015 | 0.033 | 0.46 | 0.6449 |
| Thwarted belongingness X RY vs. NRY | -0.048 | 0.019 | -2.60 | 0.0095 |

CC=Community control; RY=Remitted youth; NRY=Non-remitted youth; shading indicates passed FDR correction for interactions with remission group

**Supplemental Table S5. Details of Multilevel Models of E-Survey Positive Affect with Interaction between E-Survey Risk Factors and Final Assessment Wave Remission Group (N=159)**

|  | **DV = Positive Affect** | | | |
| --- | --- | --- | --- | --- |
| **IV = Parent Criticism/Conflict** | **Estimate** | **SE** | **t** | **p** |
| Intercept | 60.339 | 2.384 | 25.31 | <0.0001 |
| Age | -1.038 | 1.302 | -0.80 | 0.4268 |
| Wave 1 vs. Wave 2 | 0.075 | 0.360 | 0.21 | 0.8350 |
| Female sex | 1.148 | 2.430 | 0.47 | 0.6374 |
| Parent criticism/conflict | -0.184 | 0.015 | -12.22 | <0.0001 |
| CC vs. NRY | 9.130 | 3.127 | 2.92 | 0.0040 |
| RY vs. NRY | 6.701 | 2.714 | 2.47 | 0.0147 |
| Parent criticism/conflict X CC vs. NRY | -0.017 | 0.038 | -0.45 | 0.6537 |
| Parent criticism/conflict X RY vs. NRY | 0.033 | 0.022 | 1.49 | 0.1364 |
| **IV = Peer Criticism/Conflict** | **Estimate** | **SE** | **t** | **p** |
| Intercept | 60.466 | 2.382 | 25.38 | <0.0001 |
| Age | -0.633 | 1.286 | -0.49 | 0.6233 |
| Wave 1 vs. Wave 2 | -0.065 | 0.366 | -0.18 | 0.8582 |
| Female sex | 1.011 | 2.429 | 0.42 | 0.6779 |
| Peer criticism/conflict | -0.096 | 0.015 | -6.33 | <0.0001 |
| CC vs. NRY | 9.274 | 3.125 | 2.97 | 0.0035 |
| RY vs. NRY | 6.647 | 2.712 | 2.45 | 0.0154 |
| Peer criticism/conflict X CC vs. NRY | -0.031 | 0.039 | -0.80 | 0.4230 |
| Peer criticism/conflict X RY vs. NRY | -0.005 | 0.021 | -0.24 | 0.8074 |
| **IV = Perceived Burdensomeness** | **Estimate** | **SE** | **t** | **p** |
| Intercept | 60.244 | 2.373 | 25.38 | <0.0001 |
| Age | -0.162 | 1.272 | -0.13 | 0.8989 |
| Wave 1 vs. Wave 2 | 0.054 | 0.358 | 0.15 | 0.8803 |
| Female sex | 1.287 | 2.419 | 0.53 | 0.5953 |
| Perceived burdensomeness | -0.195 | 0.015 | -12.84 | <0.0001 |
| CC vs. NRY | 9.379 | 3.113 | 3.01 | 0.0030 |
| RY vs. NRY | 6.710 | 2.700 | 2.48 | 0.0141 |
| Perceived burdensomeness X CC vs. NRY | 0.083 | 0.050 | 1.66 | 0.0968 |
| Perceived burdensomeness X RY vs. NRY | 0.020 | 0.022 | 0.91 | 0.3611 |
| **IV = Thwarted Belongingness** | **Estimate** | **SE** | **t** | **p** |
| Intercept | 60.534 | 2.362 | 25.63 | <0.0001 |
| Age | -0.411 | 1.207 | -0.34 | 0.7341 |
| Wave 1 vs. Wave 2 | -0.511 | 0.351 | -1.46 | 0.1450 |
| Female sex | 1.616 | 2.413 | 0.67 | 0.5041 |
| Thwarted belongingness | -0.322 | 0.016 | -20.15 | <0.0001 |
| CC vs. NRY | 9.150 | 3.100 | 2.95 | 0.0036 |
| RY vs. NRY | 6.914 | 2.694 | 2.57 | 0.0112 |
| Thwarted belongingness X CC vs. NRY | 0.057 | 0.037 | 1.53 | 0.1252 |
| Thwarted belongingness X RY vs. NRY | 0.107 | 0.021 | 5.04 | <0.0001 |

CC=Community control; RY=Remitted youth; NRY=Non-remitted youth

**Supplemental Table S6. Details of Multilevel Models of EARS Positive Affect, Negative Affect, and Connectedness by Wave 2 Remission Group and Conflicts with Friends and Family at the Same Prompt (N=53)**

|  | **DV = Negative Affect** | | | |
| --- | --- | --- | --- | --- |
| **M1: IV = Friend Conflicts** | **Estimate** | **SE** | **t** | **p** |
| Intercept | 14.770 | 2.258 | 6.54 | <0.0001 |
| Hour of day | 0.062 | 0.096 | 0.65 | 0.5179 |
| Friend conflicts | 0.196 | 0.051 | 3.87 | 0.0001 |
| **M2: IV = Friend Conflicts** | **Estimate** | **SE** | **t** | **p** |
| Intercept | 21.661 | 3.348 | 6.47 | <0.0001 |
| Hour of day | 0.064 | 0.102 | 0.62 | 0.5345 |
| CC vs. NRY | -10.136 | 4.966 | -2.04 | 0.0468 |
| RY vs. NRY | -10.930 | 3.950 | -2.77 | 0.0079 |
| Friend conflicts | 0.221 | 0.062 | 3.56 | 0.0004 |
| Friend conflicts X CC vs. NRY | -0.203 | 0.258 | -0.79 | 0.4316 |
| Friend conflicts X RY vs. NRY | -0.079 | 0.109 | -0.73 | 0.4673 |
| **M1: IV = Family Conflicts** | **Estimate** | **SE** | **t** | **p** |
| Intercept | 14.612 | 2.139 | 6.83 | <0.0001 |
| Hour of day | 0.062 | 0.090 | 0.69 | 0.4914 |
| Family conflicts | 0.392 | 0.033 | 11.75 | <0.0001 |
| **M2: IV = Family Conflicts** | **Estimate** | **SE** | **t** | **p** |
| Intercept | 20.862 | 3.246 | 6.43 | <0.0001 |
| Hour of day | 0.082 | 0.097 | 0.85 | 0.3976 |
| CC vs. NRY | -9.800 | 4.813 | -2.04 | 0.0474 |
| RY vs. NRY | -10.258 | 3.841 | -2.67 | 0.0102 |
| Family conflicts | 0.424 | 0.051 | 8.35 | <0.0001 |
| Family conflicts X CC vs. NRY | -0.210 | 0.111 | -1.89 | 0.0592 |
| Family conflicts X RY vs. NRY | -0.139 | 0.082 | -1.70 | 0.0896 |
|  | **DV = Connectedness** | | | |
| **M1: IV = Friend Conflicts** | **Estimate** | **SE** | **t** | **p** |
| Intercept | 72.986 | 2.782 | 26.24 | <0.0001 |
| Hour of day | 0.471 | 0.106 | 4.44 | <0.0001 |
| Friend conflicts | -0.217 | 0.056 | -3.84 | 0.0001 |
| **M2: IV = Friend Conflicts** | **Estimate** | **SE** | **t** | **p** |
| Intercept | 61.429 | 3.946 | 15.57 | <0.0001 |
| Hour of day | 0.424 | 0.110 | 3.86 | 0.0001 |
| CC vs. NRY | 18.098 | 6.009 | 3.01 | 0.0041 |
| RY vs. NRY | 19.576 | 4.778 | 4.10 | 0.0002 |
| Friend conflicts | -0.269 | 0.068 | -3.96 | <0.0001 |
| Friend conflicts X CC vs. NRY | 0.178 | 0.282 | 0.63 | 0.5281 |
| Friend conflicts X RY vs. NRY | 0.144 | 0.118 | 1.22 | 0.2240 |

CC=Community control; RY=Remitted youth; NRY=Non-remitted youth

**Supplemental Table S6. Details of Multilevel Models of EARS Positive Affect, Negative Affect, and Connectedness by Wave 2 Remission Group and Conflicts with Friends and Family at the Same Prompt (N=53) (Continued, page 2)**

|  | **DV = Connectedness** | | | |
| --- | --- | --- | --- | --- |
| **M1: IV = Family Conflicts** | **Estimate** | **SE** | **t** | **p** |
| Intercept | 73.311 | 2.738 | 26.78 | <0.0001 |
| Hour of day | 0.456 | 0.104 | 4.39 | <0.0001 |
| Family conflicts | -0.308 | 0.040 | -7.77 | <0.0001 |
| **M2: IV = Family Conflicts** | **Estimate** | **SE** | **t** | **p** |
| Intercept | 62.256 | 3.936 | 15.82 | <0.0001 |
| Hour of day | 0.388 | 0.109 | 3.55 | 0.0004 |
| CC vs. NRY | 18.080 | 5.963 | 3.03 | 0.0039 |
| RY vs. NRY | 19.240 | 4.756 | 4.05 | 0.0002 |
| Family conflicts | -0.375 | 0.059 | -6.33 | <0.0001 |
| Family conflicts X CC vs. NRY | 0.220 | 0.129 | 1.70 | 0.0903 |
| Family conflicts X RY vs. NRY | 0.262 | 0.095 | 2.75 | 0.0061 |
|  | **DV = Positive Affect** | | | |
| **M1: IV = Friend Conflicts** | **Estimate** | **SE** | **t** | **p** |
| Intercept | 54.142 | 3.291 | 16.45 | <0.0001 |
| Hour of day | 0.641 | 0.124 | 5.17 | <0.0001 |
| Friend conflicts | -0.113 | 0.066 | -1.70 | 0.0896 |
| **M2: IV = Friend Conflicts** | **Estimate** | **SE** | **t** | **p** |
| Intercept | 43.189 | 4.874 | 8.86 | <0.0001 |
| Hour of day | 0.565 | 0.132 | 4.29 | <0.0001 |
| CC vs. NRY | 18.101 | 7.480 | 2.42 | 0.0193 |
| RY vs. NRY | 18.659 | 5.942 | 3.14 | 0.0028 |
| Friend conflicts | -0.169 | 0.082 | -2.05 | 0.0404 |
| Friend conflicts X CC vs. NRY | 0.472 | 0.340 | 1.39 | 0.1655 |
| Friend conflicts X RY vs. NRY | 0.133 | 0.143 | 0.93 | 0.3507 |
| **M1: IV = Family Conflicts** | **Estimate** | **SE** | **t** | **p** |
| Intercept | 54.028 | 3.235 | 16.70 | <0.0001 |
| Hour of day | 0.661 | 0.121 | 5.48 | <0.0001 |
| Family conflicts | -0.343 | 0.047 | -7.31 | <0.0001 |
| **M2: IV = Family Conflicts** | **Estimate** | **SE** | **t** | **p** |
| Intercept | 43.633 | 4.843 | 9.01 | <0.0001 |
| Hour of day | 0.569 | 0.130 | 4.39 | <0.0001 |
| CC vs. NRY | 17.111 | 7.407 | 2.31 | 0.0253 |
| RY vs. NRY | 18.054 | 5.901 | 3.06 | 0.0036 |
| Family conflicts | -0.385 | 0.072 | -5.38 | <0.0001 |
| Family conflicts X CC vs. NRY | 0.236 | 0.157 | 1.50 | 0.1346 |
| Family conflicts X RY vs. NRY | 0.113 | 0.115 | 0.98 | 0.3266 |

CC=Community control; RY=Remitted youth; NRY=Non-remitted youth; shading indicates passed FDR correction for interaction with remission group

**Supplemental Table S7. Details of Multilevel Models of E-Survey Depression, Suicidal Ideation, Negative Affect, and Positive Affect with Parent/Peer Conflict at Prior Week (N=137)**

|  | **DV = Depression at *t+1*** | | | |
| --- | --- | --- | --- | --- |
| **IV = Parent Criticism/Conflict at *t*** | **Estimate** | **SE** | **t** | **p** |
| Intercept | 0.225 | 0.026 | 8.52 | <0.0001 |
| Age | 0.012 | 0.010 | 1.27 | 0.2065 |
| Wave 1 vs. Wave 2 | 0.007 | 0.016 | 0.44 | 0.6573 |
| Female sex | 0.125 | 0.045 | 2.80 | 0.0058 |
| Depression at *t* | 0.366 | 0.012 | 31.14 | <0.0001 |
| Parent criticism/conflict at *t* | 0.000 | 0.000 | 1.72 | 0.0859 |
| **IV = Peer Criticism/Conflict at *t*** | **Estimate** | **SE** | **t** | **p** |
| Intercept | 0.226 | 0.026 | 8.58 | <0.0001 |
| Age | 0.011 | 0.010 | 1.14 | 0.2568 |
| Wave 1 vs. Wave 2 | 0.005 | 0.016 | 0.32 | 0.7520 |
| Female sex | 0.125 | 0.045 | 2.80 | 0.0059 |
| Depression at *t* | 0.369 | 0.012 | 32.04 | <0.0001 |
| Peer criticism/conflict at *t* | 0.000 | 0.000 | 1.04 | 0.2997 |
|  | **DV = Suicidal Ideation at *t+1*** | | | |
| **IV = Parent Criticism/Conflict at *t*** | **Estimate** | **SE** | **t** | **p** |
| Intercept | -5.608 | 0.453 | -12.38 | <0.0001 |
| Age | -0.254 | 0.253 | -1.00 | 0.3168 |
| Wave 1 vs. Wave 2 | 0.110 | 0.485 | 0.23 | 0.8207 |
| Female sex | 2.533 | 0.507 | 4.99 | <0.0001 |
| Suicidal ideation at *t* | 1.066 | 0.172 | 6.19 | <0.0001 |
| Parent criticism/conflict at *t* | 0.009 | 0.003 | 2.51 | 0.0120 |
| **IV = Peer Criticism/Conflict at *t*** | **Estimate** | **SE** | **t** | **p** |
| Intercept | -5.584 | 0.453 | -12.34 | <0.0001 |
| Age | -0.288 | 0.253 | -1.14 | 0.2569 |
| Wave 1 vs. Wave 2 | 0.070 | 0.484 | 0.14 | 0.8847 |
| Female sex | 2.532 | 0.508 | 4.99 | <0.0001 |
| Suicidal ideation at *t* | 1.109 | 0.172 | 6.45 | <0.0001 |
| Peer criticism/conflict at *t* | 0.005 | 0.003 | 1.40 | 0.1602 |
|  | **DV = Negative Affect at *t+1*** | | | |
| **IV = Parent Criticism/Conflict at *t*** | **Estimate** | **SE** | **t** | **p** |
| Intercept | 27.699 | 1.695 | 16.34 | <0.0001 |
| Age | -1.560 | 0.631 | -2.47 | 0.0144 |
| Wave 1 vs. Wave 2 | -2.047 | 1.027 | -1.99 | 0.0463 |
| Female sex | 8.545 | 2.914 | 2.93 | 0.0040 |
| Negative affect at *t* | 0.331 | 0.012 | 28.16 | <0.0001 |
| Parent criticism/conflict at *t* | 0.052 | 0.010 | 5.36 | <0.0001 |
| **IV = Peer Criticism/Conflict at *t*** | **Estimate** | **SE** | **t** | **p** |
| Intercept | 27.840 | 1.694 | 16.43 | <0.0001 |
| Age | -1.782 | 0.634 | -2.81 | 0.0056 |
| Wave 1 vs. Wave 2 | -2.284 | 1.025 | -2.23 | 0.0259 |
| Female sex | 8.495 | 2.914 | 2.92 | 0.0042 |
| Negative affect at *t* | 0.337 | 0.011 | 29.30 | <0.0001 |
| Peer criticism/conflict at *t* | 0.039 | 0.009 | 4.23 | <0.0001 |

**Supplemental Table S7. Details of Multilevel Models of E-Survey Depression, Suicidal Ideation, Negative Affect, and Positive Affect with Parent/Peer Conflict at Prior Week (N=137) (Continued, page 2)**

|  | **DV = Positive Affect at *t+1*** | | | |
| --- | --- | --- | --- | --- |
| **IV = Parent Criticism/Conflict at *t*** | **Estimate** | **SE** | **t** | **p** |
| Intercept | 64.404 | 1.800 | 35.81 | <0.0001 |
| Age | -0.219 | 0.790 | -0.28 | 0.7823 |
| Wave 1 vs. Wave 2 | -0.150 | 1.119 | -0.13 | 0.8934 |
| Female sex | -1.657 | 3.082 | -0.54 | 0.5918 |
| Positive affect at *t* | 0.300 | 0.012 | 25.69 | <0.0001 |
| Parent criticism/conflict at *t* | -0.036 | 0.010 | -3.55 | 0.0004 |
| **IV = Peer Criticism/Conflict at *t*** | **Estimate** | **SE** | **t** | **p** |
| Intercept | 64.312 | 1.800 | 35.79 | <0.0001 |
| Age | -0.068 | 0.783 | -0.09 | 0.9312 |
| Wave 1 vs. Wave 2 | -0.002 | 1.115 | -0.00 | 0.9986 |
| Female sex | -1.595 | 3.081 | -0.52 | 0.6055 |
| Positive affect at *t* | 0.303 | 0.012 | 26.25 | <0.0001 |
| Peer criticism/conflict at *t* | -0.021 | 0.010 | -2.14 | 0.0326 |

passed FDR correction for parent or peer criticism at time *t* predicting the DV at *t+1* with the DV at *t* in the model

**Supplemental Table S8. Details of Multilevel Models of E-Survey Parent/Peer Conflict with Depression, Suicidal Ideation, Negative Affect, and Positive Affect at Prior Week (N=137)**

|  | **DV = Parent Criticism/Conflict at *t+1*** | | | |
| --- | --- | --- | --- | --- |
| **IV = Depression at *t*** | **Estimate** | **SE** | **t** | **p** |
| Intercept | 22.162 | 1.787 | 12.40 | <0.0001 |
| Age | -2.464 | 0.876 | -2.81 | 0.0059 |
| Wave 1 vs. Wave 2 | -4.698 | 1.282 | -3.67 | 0.0003 |
| Female sex | 2.214 | 2.981 | 0.74 | 0.4590 |
| Parent criticism/conflict at *t* | 0.238 | 0.012 | 19.56 | <0.0001 |
| Depression at *t* | 4.557 | 0.931 | 4.90 | <0.0001 |
| **IV = Suicidal Ideation at *t*** | **Estimate** | **SE** | **t** | **p** |
| Intercept | 21.951 | 1.785 | 12.30 | <0.0001 |
| Age | -2.233 | 0.854 | -2.62 | 0.0102 |
| Wave 1 vs. Wave 2 | -4.351 | 1.281 | -3.40 | 0.0007 |
| Female sex | 2.323 | 2.980 | 0.78 | 0.4370 |
| Parent criticism/conflict at *t* | 0.255 | 0.012 | 21.88 | <0.0001 |
| Suicidal ideation at *t* | 0.418 | 0.878 | 0.48 | 0.6340 |
| **IV = Negative Affect at *t*** | **Estimate** | **SE** | **t** | **p** |
| Intercept | 21.931 | 1.788 | 12.27 | <0.0001 |
| Age | -2.113 | 0.848 | -2.49 | 0.0143 |
| Wave 1 vs. Wave 2 | -4.317 | 1.277 | -3.38 | 0.0007 |
| Female sex | 2.294 | 2.987 | 0.77 | 0.4438 |
| Parent criticism/conflict at *t* | 0.239 | 0.012 | 19.74 | <0.0001 |
| Negative affect at *t* | 0.080 | 0.015 | 5.45 | <0.0001 |
| **IV = Positive Affect at *t*** | **Estimate** | **SE** | **t** | **p** |
| Intercept | 22.080 | 1.786 | 12.36 | <0.0001 |
| Age | -2.354 | 0.875 | -2.69 | 0.0083 |
| Wave 1 vs. Wave 2 | -4.574 | 1.281 | -3.57 | 0.0004 |
| Female sex | 2.272 | 2.981 | 0.76 | 0.4473 |
| Parent criticism/conflict at *t* | 0.247 | 0.012 | 21.10 | <0.0001 |
| Positive affect at *t* | -0.059 | 0.014 | -4.33 | <0.0001 |
|  | **DV = Peer Criticism/Conflict at *t+1*** | | | |
| **IV = Depression at *t*** | **Estimate** | **SE** | **t** | **p** |
| Intercept | 16.821 | 1.732 | 9.71 | <0.0001 |
| Age | 1.052 | 0.697 | 1.51 | 0.1331 |
| Wave 1 vs. Wave 2 | -0.889 | 1.248 | -0.71 | 0.4761 |
| Female sex | -1.132 | 2.872 | -0.39 | 0.6941 |
| Peer criticism/conflict at *t* | 0.362 | 0.012 | 31.33 | <0.0001 |
| Depression at *t* | 2.308 | 0.908 | 2.54 | 0.0110 |
| **IV = Suicidal Ideation at *t*** | **Estimate** | **SE** | **t** | **p** |
| Intercept | 16.699 | 1.731 | 9.65 | <0.0001 |
| Age | 1.132 | 0.692 | 1.64 | 0.1037 |
| Wave 1 vs. Wave 2 | -0.702 | 1.249 | -0.56 | 0.5743 |
| Female sex | -1.124 | 2.862 | -0.39 | 0.6951 |
| Peer criticism/conflict at *t* | 0.370 | 0.011 | 32.82 | <0.0001 |
| Suicidal ideation at *t* | 0.656 | 0.875 | 0.75 | 0.4537 |

**Supplemental Table S8. Details of Multilevel Models of E-Survey Parent/Peer Conflict with Depression, Suicidal Ideation, Negative Affect, and Positive Affect at Prior Week (N=137) (Continued, page 2)**

|  | **DV = Peer Criticism/Conflict at *t+1*** | | | |
| --- | --- | --- | --- | --- |
| **IV = Negative Affect at *t*** | **Estimate** | **SE** | **t** | **p** |
| Intercept | 16.694 | 1.737 | 9.61 | <0.0001 |
| Age | 1.241 | 0.692 | 1.79 | 0.0750 |
| Wave 1 vs. Wave 2 | -0.681 | 1.247 | -0.55 | 0.5852 |
| Female sex | -1.041 | 2.880 | -0.36 | 0.7183 |
| Peer criticism/conflict at *t* | 0.360 | 0.012 | 31.31 | <0.0001 |
| Negative affect at *t* | 0.061 | 0.014 | 4.25 | <0.0001 |
| **IV = Positive Affect at *t*** | **Estimate** | **SE** | **t** | **p** |
| Intercept | 16.854 | 1.735 | 9.71 | <0.0001 |
| Age | 1.068 | 0.702 | 1.52 | 0.1299 |
| Wave 1 vs. Wave 2 | -0.935 | 1.249 | -0.75 | 0.4541 |
| Female sex | -1.078 | 2.874 | -0.38 | 0.7082 |
| Peer criticism/conflict at *t* | 0.367 | 0.011 | 32.49 | <0.0001 |
| Positive affect at *t* | --0.035 | 0.013 | -2.59 | 0.0095 |

passed FDR correction for IV at time *t* predicting the peer or parent criticism at *t+1* with peer or parent criticism at time *t* in the model

**Supplemental Table S9. Details of Multilevel Models of EARS Negative Affect, Connectedness, and Positive Affect by Wave 2 Remission Group and Conflicts with Friends and Family at the Prior Prompt From the Same Day (N=53)**

|  | **DV = Negative Affect at *t+1*** | | | |
| --- | --- | --- | --- | --- |
| **M1: IV = Friend Conflicts at *t*** | **Estimate** | **SE** | **t** | **p** |
| Intercept | 15.690 | 4.725 | 3.32 | 0.0010 |
| Hour of day | -0.111 | 0.258 | -0.43 | 0.6684 |
| Negative affect at *t* | 0.354 | 0.054 | 6.58 | <0.0001 |
| Friend conflicts at *t* | 0.194 | 0.070 | 2.76 | 0.0062 |
| **M2: IV = Friend Conflicts at *t*** | **Estimate** | **SE** | **t** | **p** |
| Intercept | 18.846 | 5.130 | 3.67 | 0.0003 |
| Hour of day | 0.035 | 0.257 | 0.13 | 0.8931 |
| CC vs. NRY | -8.547 | 3.815 | -2.24 | 0.0329 |
| RY vs. NRY | -8.646 | 3.105 | -2.78 | 0.0089 |
| Negative affect at *t* | 0.266 | 0.059 | 4.51 | <0.0001 |
| Friend conflicts at *t* | 0.198 | 0.078 | 2.53 | 0.0122 |
| Friend conflicts at *t* X CC vs. NRY | 0.572 | 0.308 | 1.86 | 0.0648 |
| Friend conflicts at *t* X RY vs. NRY | -0.121 | 0.154 | -0.79 | 0.4325 |
| **M1: IV = Family Conflicts at *t*** | **Estimate** | **SE** | **t** | **p** |
| Intercept | 14.792 | 4.745 | 3.12 | 0.0020 |
| Hour of day | -0.050 | 0.259 | -0.19 | 0.8468 |
| Negative affect at *t* | 0.345 | 0.057 | 6.03 | <0.0001 |
| Family conflicts at *t* | 0.113 | 0.061 | 1.85 | 0.0647 |
| **M2: IV = Family Conflicts at *t*** | **Estimate** | **SE** | **t** | **p** |
| Intercept | 16.749 | 5.205 | 3.22 | 0.0015 |
| Hour of day | 0.157 | 0.259 | 0.61 | 0.5437 |
| CC vs. NRY | -9.995 | 3.848 | -2.60 | 0.0144 |
| RY vs. NRY | -8.713 | 3.142 | -2.77 | 0.0090 |
| Negative affect at *t* | 0.268 | 0.063 | 4.27 | <0.0001 |
| Family conflicts at *t* | 0.234 | 0.088 | 2.65 | 0.0087 |
| Family conflicts X CC vs. NRY | -0.212 | 0.229 | -0.93 | 0.3539 |
| Family conflicts X RY vs. NRY | -0.357 | 0.122 | -2.94 | 0.0037 |
|  | **DV = Connectedness at *t+1*** | | | |
| **M1: IV = Friend Conflicts at *t*** | **Estimate** | **SE** | **t** | **p** |
| Intercept | 78.009 | 6.103 | 12.78 | <0.0001 |
| Hour of day | 0.169 | 0.331 | 0.51 | 0.6096 |
| Connectedness at *t* | 0.374 | 0.058 | 6.48 | <0.0001 |
| Friend conflicts at *t* | -0.051 | 0.090 | -0.57 | 0.5724 |
| **M2: IV = Friend Conflicts at *t*** | **Estimate** | **SE** | **t** | **p** |
| Intercept | 65.282 | 6.739 | 9.69 | <0.0001 |
| Hour of day | 0.201 | 0.323 | 0.62 | 0.5339 |
| CC vs. NRY | 19.831 | 5.322 | 3.73 | 0.0006 |
| RY vs. NRY | 18.652 | 4.377 | 4.26 | 0.0001 |
| Connectedness at *t* | 0.287 | 0.061 | 4.74 | <0.0001 |
| Friend conflicts at *t* | 0.010 | 0.097 | 0.10 | 0.9219 |
| Friend conflicts at *t* X CC vs. NRY | 0.408 | 0.384 | 1.06 | 0.2894 |
| Friend conflicts at *t* X RY vs. NRY | -0.240 | 0.193 | -1.24 | 0.2150 |

CC=Community control; RY=Remitted youth; NRY=Non-remitted youth; shading indicates passed FDR correction for interaction with remission group

**Supplemental Table S9. Details of Multilevel Models of EARS Negative Affect, Connectedness, and Positive Affect by Wave 2 Remission Group and Conflicts with Friends and Family at the Prior Prompt From the Same Day (Continued, page 2)**

|  | **DV = Connectedness at *t+1*** | | | |
| --- | --- | --- | --- | --- |
| **M1: IV = Family Conflicts at *t*** | **Estimate** | **SE** | **t** | **p** |
| Intercept | 77.982 | 5.969 | 13.06 | <0.0001 |
| Hour of day | 0.176 | 0.320 | 0.55 | 0.5823 |
| Connectedness at *t* | 0.306 | 0.058 | 5.27 | <0.0001 |
| Family conflicts at *t* | -0.226 | 0.072 | -3.14 | 0.0019 |
| **M2: IV = Family Conflicts at *t*** | **Estimate** | **SE** | **t** | **p** |
| Intercept | 66.307 | 6.723 | 9.86 | <.0001 |
| Hour of day | 0.184 | 0.313 | 0.59 | 0.5579 |
| CC vs. NRY | 19.104 | 5.652 | 3.38 | 0.0018 |
| RY vs. NRY | 18.651 | 4.624 | 4.03 | 0.0002 |
| Connectedness at *t* | 0.205 | 0.062 | 3.30 | 0.0011 |
| Family conflicts at *t* | -0.349 | 0.107 | -3.25 | 0.0013 |
| Family conflicts at *t* X CC vs. NRY | 0.311 | 0.277 | 1.12 | 0.2634 |
| Family conflicts at *t* X RY vs. NRY | 0.383 | 0.151 | 2.55 | 0.0116 |
|  | **DV = Positive Affect at *t+1*** | | | |
| **M1: IV = Friend Conflicts at *t*** | **Estimate** | **SE** | **t** | **p** |
| Intercept | 61.565 | 6.352 | 9.69 | <0.0001 |
| Hour of day | 0.267 | 0.339 | 0.79 | 0.4326 |
| Positive affect at *t* | 0.387 | 0.052 | 7.42 | <0.0001 |
| Friend conflicts at *t* | 0.060 | 0.093 | 0.64 | 0.5230 |
| **M2: IV = Friend Conflicts at *t*** | **Estimate** | **SE** | **t** | **p** |
| Intercept | 51.419 | 7.645 | 6.73 | <0.0001 |
| Hour of day | 0.268 | 0.361 | 0.74 | 0.4592 |
| CC vs. NRY | 14.571 | 6.541 | 2.23 | 0.0332 |
| RY vs. NRY | 15.307 | 5.315 | 2.88 | 0.0068 |
| Positive affect at *t* | 0.375 | 0.057 | 6.54 | <0.0001 |
| Friend conflicts at *t* | 0.146 | 0.112 | 1.31 | 0.1913 |
| Friend conflicts at *t* X CC vs. NRY | -0.686 | 0.430 | -1.59 | 0.1126 |
| Friend conflicts at *t* X RY vs. NRY | -0.091 | 0.218 | -0.42 | 0.6762 |
| **M1: IV = Family Conflicts at *t*** | **Estimate** | **SE** | **t** | **p** |
| Intercept | 62.279 | 6.375 | 9.77 | <0.0001 |
| Hour of day | 0.227 | 0.340 | 0.67 | 0.5039 |
| Positive affect at *t* | 0.367 | 0.054 | 6.86 | <0.0001 |
| Family conflicts at *t* | -0.045 | 0.077 | -0.59 | 0.5578 |
| **M2: IV = Family Conflicts at *t*** | **Estimate** | **SE** | **t** | **p** |
| Intercept | 53.938 | 7.777 | 6.94 | <0.0001 |
| Hour of day | 0.200 | 0.364 | 0.55 | 0.5837 |
| CC vs. NRY | 14.561 | 6.537 | 2.23 | 0.0327 |
| RY vs. NRY | 13.913 | 5.333 | 2.61 | 0.0129 |
| Positive affect at *t* | 0.353 | 0.059 | 5.99 | <0.0001 |
| Family conflicts at *t* | -0.119 | 0.122 | -0.97 | 0.3328 |
| Family conflicts at *t* X CC vs. NRY | 0.198 | 0.324 | 0.61 | 0.5413 |
| Family conflicts at *t* X RY vs. NRY | 0.211 | 0.173 | 1.22 | 0.2256 |

CC=Community control; RY=Remitted youth; NRY=Non-remitted youth; shading indicates passed FDR correction

**Supplemental Table S10. Details of Multilevel Models of EARS Friend Conflicts and Family Conflicts by Wave 2 Remission Group and Negative Affect, Positive Affect, and Connectedness at the Prior Prompt From the Same Day (N=53)**

|  | **DV = Family Conflicts at *t+1*** | | | |
| --- | --- | --- | --- | --- |
| **M1: IV = Negative Affect at *t*** | **Estimate** | **SE** | **t** | **p** |
| Intercept | -1.791 | 3.711 | -0.48 | 0.6300 |
| Hour of day | 0.296 | 0.202 | 1.46 | 0.1456 |
| Family conflicts at *t* | 0.043 | 0.052 | 0.82 | 0.4148 |
| Negative affect at *t* | 0.107 | 0.045 | 2.36 | 0.0193 |
| **M2: IV = Negative Affect at *t*** | **Estimate** | **SE** | **t** | **p** |
| Intercept | 0.289 | 4.148 | 0.07 | 0.9445 |
| Hour of day | 0.243 | 0.214 | 1.14 | 0.2568 |
| CC vs. NRY | -0.528 | 2.400 | -0.22 | 0.8272 |
| RY vs. NRY | -3.276 | 1.979 | -1.66 | 0.1071 |
| Family conflicts at *t* | -0.040 | 0.054 | -0.73 | 0.4642 |
| Negative affect at *t* | 0.065 | 0.067 | 0.98 | 0.3279 |
| Negative affect at *t* X CC vs. NRY | 0.031 | 0.150 | 0.21 | 0.8348 |
| Negative affect at *t* X RY vs. NRY | -0.014 | 0.109 | -0.12 | 0.9007 |
| **M1: IV = Positive Affect at *t*** | **Estimate** | **SE** | **t** | **p** |
| Intercept | -2.673 | 3.737 | -0.72 | 0.4755 |
| Hour of day | 0.345 | 0.203 | 1.70 | 0.0919 |
| Family conflicts at *t* | 0.074 | 0.050 | 1.47 | 0.1432 |
| Positive affect at *t* | -0.042 | 0.031 | -1.35 | 0.1777 |
| **M2: IV = Positive Affect at *t*** | **Estimate** | **SE** | **t** | **p** |
| Intercept | -0.191 | 4.165 | -0.05 | 0.9635 |
| Hour of day | 0.268 | 0.214 | 1.26 | 0.2111 |
| CC vs. NRY | -0.719 | 2.376 | -0.30 | 0.7643 |
| RY vs. NRY | -3.535 | 2.078 | -1.70 | 0.0972 |
| Family conflicts at *t* | -0.021 | 0.051 | -0.41 | 0.6848 |
| Positive affect at *t* | -0.034 | 0.050 | -0.68 | 0.4978 |
| Positive affect at *t* X CC vs. NRY | -0.014 | 0.085 | -0.16 | 0.8739 |
| Positive affect at *t* X RY vs. NRY | 0.046 | 0.076 | 0.60 | 0.5495 |
| **M1: IV = Connectedness at *t*** | **Estimate** | **SE** | **t** | **p** |
| Intercept | -3.158 | 3.680 | -0.86 | 0.3922 |
| Hour of day | 0.377 | 0.201 | 1.88 | 0.0626 |
| Family conflicts at *t* | 0.065 | 0.049 | 1.32 | 0.1879 |
| Connectedness at *t* | -0.087 | 0.033 | -2.60 | 0.0103 |
| **M2: IV = Connectedness at *t*** | **Estimate** | **SE** | **t** | **p** |
| Intercept | -2.403 | 4.095 | -0.59 | 0.5581 |
| Hour of day | 0.336 | 0.210 | 1.60 | 0.1126 |
| CC vs. NRY | 1.492 | 2.281 | 0.65 | 0.5140 |
| RY vs. NRY | -1.915 | 1.923 | -1.00 | 0.3207 |
| Family conflicts at *t* | -0.029 | 0.050 | -0.59 | 0.5579 |
| Connectedness at *t* | -0.098 | 0.048 | -2.05 | 0.0417 |
| Connectedness at *t* X CC vs. NRY | -0.061 | 0.102 | -0.60 | 0.5523 |
| Connectedness at *t* X RY vs. NRY | 0.040 | 0.083 | 0.48 | 0.6325 |

CC=Community control; RY=Remitted youth; NRY=Non-remitted youth

**Supplemental Table S10. Details of Multilevel Models of EARS Friend Conflicts and Family Conflicts by Wave 2 Remission Group and Negative Affect, Positive Affect, and Connectedness at the Prior Prompt From the Same Day (Continued, page 2)**

|  | **DV = Friend Conflicts at *t+1*** | | | |
| --- | --- | --- | --- | --- |
| **M1: IV = Negative Affect at *t*** | **Estimate** | **SE** | **t** | **p** |
| Intercept | -4.207 | 3.439 | -1.22 | 0.2227 |
| Hour of day | 0.372 | 0.189 | 1.97 | 0.0503 |
| Friend conflicts at *t* | 0.068 | 0.052 | 1.30 | 0.1962 |
| Negative affect at *t* | -0.008 | 0.038 | -0.21 | 0.8378 |
| **M2: IV = Negative Affect at *t*** | **Estimate** | **SE** | **t** | **p** |
| Intercept | -1.854 | 4.272 | -0.43 | 0.6649 |
| Hour of day | 0.410 | 0.221 | 1.85 | 0.0659 |
| CC vs. NRY | -4.587 | 2.385 | -1.92 | 0.0628 |
| RY vs. NRY | -3.983 | 1.948 | -2.04 | 0.0489 |
| Friend conflicts at *t* | 0.060 | 0.058 | 1.02 | 0.3089 |
| Negative affect at *t* | -0.041 | 0.066 | -0.63 | 0.5279 |
| Negative affect at *t* X CC vs. NRY | -0.045 | 0.150 | -0.30 | 0.7651 |
| Negative affect at *t* X RY vs. NRY | 0.001 | 0.108 | 0.01 | 0.9947 |
| **M1: IV = Positive Affect at *t*** | **Estimate** | **SE** | **t** | **p** |
| Intercept | -4.190 | 3.422 | -1.22 | 0.2222 |
| Hour of day | 0.372 | 0.188 | 1.98 | 0.0493 |
| Friend conflicts at *t* | 0.067 | 0.051 | 1.33 | 0.1841 |
| Positive affect at *t* | -0.020 | 0.026 | -0.76 | 0.4466 |
| **M2: IV = Positive Affect at *t*** | **Estimate** | **SE** | **t** | **p** |
| Intercept | -1.655 | 4.271 | -0.39 | 0.6989 |
| Hour of day | 0.377 | 0.219 | 1.72 | 0.0876 |
| CC vs. NRY | -4.004 | 2.337 | -1.71 | 0.0985 |
| RY vs. NRY | -3.540 | 2044 | -1.73 | 0.0923 |
| Friend conflicts at *t* | 0.045 | 0.056 | 0.81 | 0.4190 |
| Positive affect at *t* | -0.014 | 0.050 | -0.29 | 0.7759 |
| Positive affect at *t* X CC vs. NRY | 0.059 | 0.084 | 0.70 | 0.4831 |
| Positive affect at *t* X RY vs. NRY | 0.025 | 0.077 | 0.33 | 0.7451 |
| **M1: IV = Connectedness at *t*** | **Estimate** | **SE** | **t** | **p** |
| Intercept | -4.276 | 3.417 | -1.25 | 0.2121 |
| Hour of day | 0.379 | 0.188 | 2.02 | 0.0453 |
| Friend conflicts at *t* | 0.064 | 0.051 | 1.27 | 0.2061 |
| Connectedness at *t* | -0.034 | 0.029 | -1.17 | 0.2424 |
| **M2: IV = Connectedness at *t*** | **Estimate** | **SE** | **t** | **p** |
| Intercept | -2.020 | 4.328 | -0.47 | 0.6413 |
| Hour of day | 0.399 | 0.221 | 1.80 | 0.0730 |
| CC vs. NRY | -3.242 | 2.440 | -1.33 | 0.1936 |
| RY vs. NRY | -3.553 | 2.053 | -1.73 | 0.0920 |
| Friend conflicts at *t* | 0.048 | 0.056 | 0.86 | 0.3906 |
| Connectedness at *t* | -0.009 | 0.051 | -0.18 | 0.8597 |
| Connectedness at *t* X CC vs. NRY | -0.030 | 0.109 | -0.27 | 0.7855 |
| Connectedness at *t* X RY vs. NRY | 0.019 | 0.088 | 0.22 | 0.8255 |

CC=Community control; RY=Remitted youth; NRY=Non-remitted youth

**Supplemental Figure S2. Thwarted Belongingness X Remission Group 🡪 Positive Affect in Concurrent Model**

**
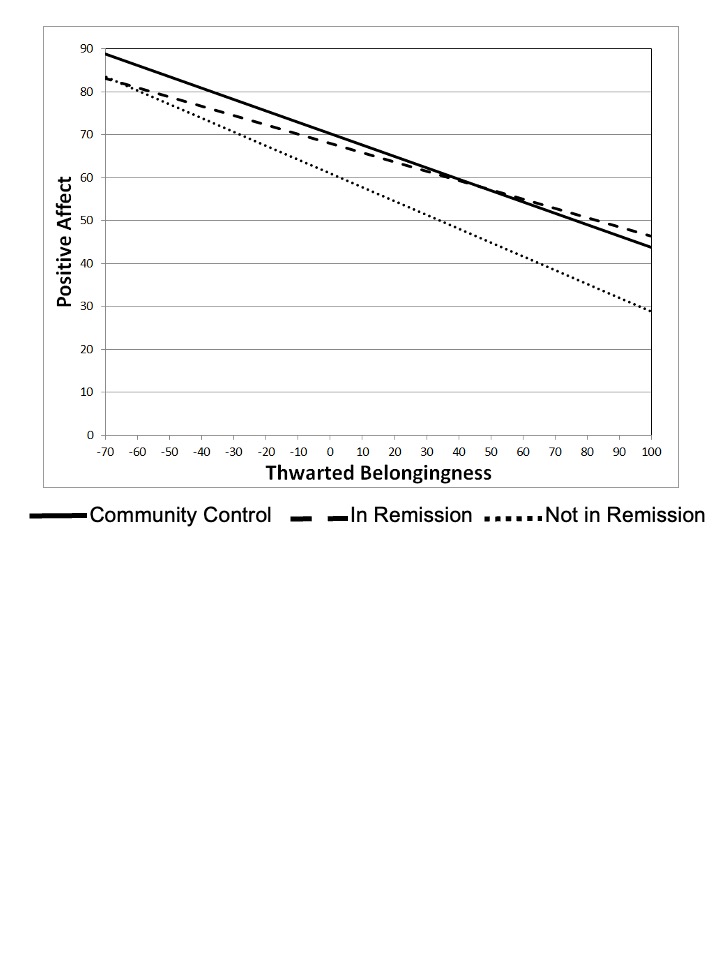
**

**Supplemental Figure S3. Family conflicts X Remission group 🡪 Connectedness in Concurrent Model**


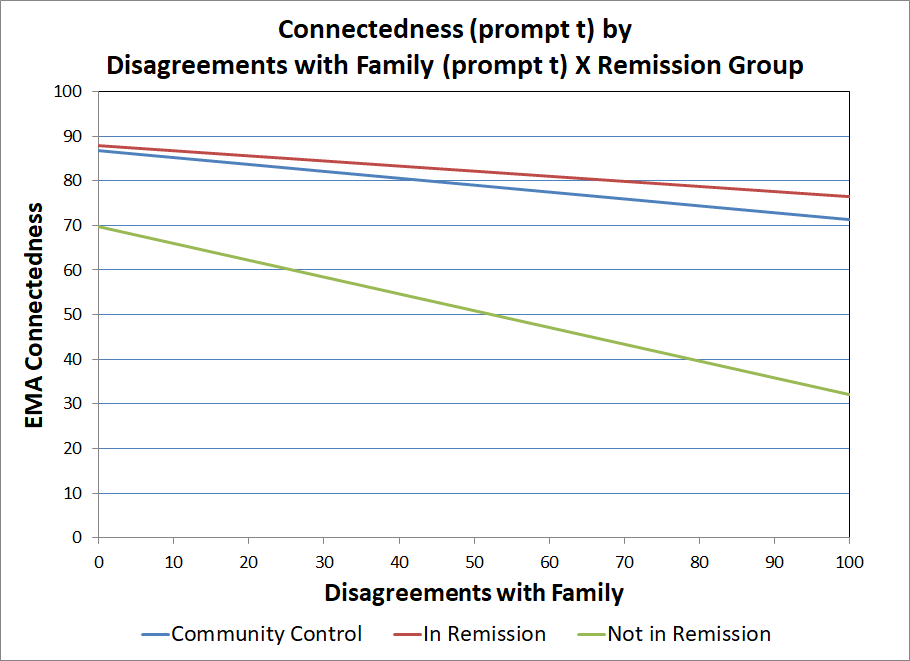

Supplement: Supplementary file 1 — Supporting Information Supplemental Methods. Table S1: Subject characteristics in those included in versus excluded from analyses. Table S2: Details of multilevel models of e‐survey depression with interaction between e‐survey risk factors and final assessment wave remission group (NN = 159). Table S3: Details of multilevel models of e‐survey suicidal ideation with interaction between e‐survey risk factors and final assessment wave remission group (NN = 159). Table S4: Details of multilevel models of e‐survey negative affect with interaction between e‐survey risk factors and final assessment wave remission group (NN = 159). Table S5: Details of multilevel models of e‐survey positive affect with interaction between e‐survey risk factors and final assessment wave remission group (NN = 159). Table S6: Details of multilevel models of EARS positive affect, negative affect, and connectedness by Wave 2 remission group and conflicts with friends and family at the same prompt (NN = 53). Table S7: Details of multilevel models of e‐survey depression, suicidal ideation, negative affect, and positive affect with parent/peer conflict at prior week (NN = 137). Table S8: Details of multilevel models of e‐survey parent/peer conflict with depression, suicidal ideation, negative affect, and positive affect at prior week (NN = 137). Table S9: Details of multilevel models of EARS negative affect, connectedness, and positive affect by Wave 2 remission group and conflicts with friends and family at the prior prompt from the same day (NN = 53). Table S10: Details of multilevel models of EARS friend conflicts and family conflicts by Wave 2 remission group and negative affect, positive affect, and connectedness at the prior prompt from the same day (NN = 53). Figure S1: Timeline of assessments and sample sizes. Figure S2: Thwarted belongingness X remission group positive affect in concurrent model. Figure S3: Family conflicts X remission group connectedness in concurrent model. [file DA-2026-5803578-s001.docx]
